# The cytosolic iron–sulfur cluster assembly (CIA) pathway is required for replication stress tolerance of cancer cells to Chk1 and ATR inhibitors

**DOI:** 10.1038/s41523-021-00353-2

**Published:** 2021-12-02

**Authors:** Abena B. Redwood, Xiaomei Zhang, Sahil B. Seth, Zhongqi Ge, Wendy E. Bindeman, Xinhui Zhou, Vidya C. Sinha, Timothy P. Heffernan, Helen Piwnica-Worms

**Affiliations:** 1 Department of Experimental Radiation Oncology, The University of Texas MD Anderson Cancer Center, Houston, TX 77030 USA; 2 Institute of Applied Cancer Science, The University of Texas MD Anderson Cancer Center, Houston, TX 77030 USA; 3 TRACTION Platform, The University of Texas MD Anderson Cancer Center, Houston, TX 77030 USA; 4 Department of Bioinformatics and Computational Biology, The University of Texas MD Anderson Cancer Center, Houston, TX 77030 USA; 5 Present Address: Vanderbilt University, Department of Cancer Biology, Nashville, TN 37235 USA

**Keywords:** Breast cancer, Breast cancer

## Abstract

The relationship between ATR/Chk1 activity and replication stress, coupled with the development of potent and tolerable inhibitors of this pathway, has led to the clinical exploration of ATR and Chk1 inhibitors (ATRi/Chk1i) as anticancer therapies for single-agent or combinatorial application. The clinical efficacy of these therapies relies on the ability to ascertain which patient populations are most likely to benefit, so there is intense interest in identifying predictive biomarkers of response. To comprehensively evaluate the components that modulate cancer cell sensitivity to replication stress induced by Chk1i, we performed a synthetic-lethal drop-out screen in a cell line derived from a patient with triple-negative breast cancer (TNBC), using a pooled barcoded shRNA library targeting ~350 genes involved in DNA replication, DNA damage repair, and cycle progression. In addition, we sought to compare the relative requirement of these genes when DNA fidelity is challenged by clinically relevant anticancer breast cancer drugs, including cisplatin and PARP1/2 inhibitors, that have different mechanisms of action. This global comparison is critical for understanding not only which agents should be used together for combinatorial therapies in breast cancer patients, but also the genetic context in which these therapies will be most effective, and when a single-agent therapy will be sufficient to provide maximum therapeutic benefit to the patient. We identified unique potentiators of response to ATRi/Chk1i and describe a new role for components of the cytosolic iron–sulfur assembly (CIA) pathway, MMS19 and CIA2B-FAM96B, in replication stress tolerance of TNBC.

## Introduction

DNA replication is a fundamental requirement of every proliferating cell, and faithful maintenance of genome stability hinges on the accuracy of this process. Each time a cell progresses to S-phase, it faces the challenge of unpacking and unwinding its chromatinized DNA to allow access to polymerases, and accurately replicating the roughly 6 billion complementary base pairs of the diploid genome^1^. This task is achieved by careful regulation of cell cycle progression, which ensures that cells have sufficient components of the replication machinery and nucleotides for production of nascent DNA^2,3^. Despite these precautions, DNA replication is invariably disrupted by endogenous and exogenous assaults that compromise replication fork progression^3^.

To mitigate the deleterious effects of disrupted fork progression, cells activate the ATR/Chk1-mediated replication stress response (RSR) pathway in response to stalled forks, which leads to cell cycle arrest, stabilization of stalled forks, and global inhibition of new origin firing^3–6^. During recovery from replication stress, ATR/Chk1 also activates the firing of local dormant origins to ensure complete replication through regions where forks have collapsed^7–10^. Failure to activate the ATR/Chk1-mediated RSR leads to uncontrolled firing of dormant origins, which accelerates the depletion of essential factors like RPA and nucleotides^4,11^. Exhaustion of these critical components gives rise to the nuclear-wide collapse of replication forks and replication catastrophe^12–14^. In addition, the inability to activate ATR/Chk1 increases the frequency of mitotic catastrophe, which can occur when cells progress into mitosis with unreplicated DNA^15^. Thus, inhibition of ATR/Chk1 can be lethal to cells that experience replication stress, and inhibitors of this pathway are being actively explored for clinical use in oncology as a single agent- or combinatorial-therapies^16–19^.

The combinatorial approaches being evaluated clinically generally involve the use of chemotherapy or targeted therapies that cause replication stress either by blocking fork progression (platinum-based agents), inhibiting dNTP production (gemcitabine), or altering the replication template by impairing DNA repair pathways (PARPi)^20–23^. Preclinical studies have demonstrated efficacy of these combinatorial strategies, and there is substantial evidence that single-agent ATRi/Chk1i can be highly effective in certain subsets of cancers. Head and neck cancers with heightened sensitivity to Chk1i were found to have higher levels of CDKN2A/p16 deletion^24^, while Chk1i-responsive small cell lung carcinoma cell lines featured increased activation of oncogenic Myc^4^. Although oncogene activation can correlate with sensitivity to ATRi/Chk1i, studies by Murga et al. demonstrated that with respect to therapeutic efficacy, not all oncogenes are created equal^16,19^. For example, Myc- but not Kras-driven pancreatic tumors xenografts could be successfully treated with Chk1i. Thus, additional studies are needed to fully understand the mechanisms that govern cancer cell response to single-agent ATRi/Chk1i, and drive the development of biomarker panels that can reliably guide treatment decisions.

In this study, we performed parallel loss-of-function synthetic-lethal screens with Chk1i as well as PARPi and cisplatin, in order to rank the genetic contexts that impart maximum sensitivity to each agent, and uncover novel pathways of dependency. Our data reveal a new relationship between the cytosolic iron–sulfur cluster assembly (CIA) pathway and cancer cell response to ATRi/Chk1i in TNBC cells.

## Results

### Targeted shRNA drop-out screens identify modulators of cancer cell sensitivity to DNA damage and replication stress

**Fig. 1:**
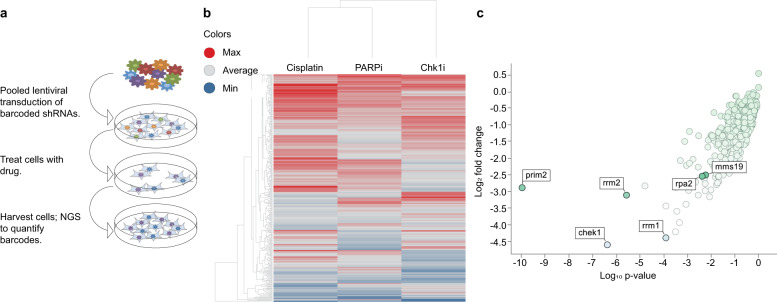
High-throughput loss of function screen to evaluate and prioritize cell cycle and DDR pathways that regulate cancer cell response to agents that induce replication stress and/or disrupt DNA fidelity. **a** Schematic of the screen: BC3-A2 TNBC cells were lentivirally transduced with a pooled barcoded shRNA library targeting ~350 genes that regulate the DNA damage response, DNA replication, and/or cell cycle progression, followed by puromycin selection. Transduced cells were plated at a density of 2.5 × 10^3^ cells per well in a 96-well format and were allowed to attach overnight (Day 0). One set of cells was treated with saline (vehicle) or 1.0 µM cisplatin (Day 1). The following day, other sets of cells were treated with either DMSO (vehicle), 10 nM LY2606368 (Chk1i), or 50 nM talazoparib (PARPi) (Day 2). On day 7, cells were washed and cultured in the presence of fresh media lacking drugs. Cells were harvested on day 14 and subjected to next-generation sequencing to quantify the frequency of each barcode. Barcode frequencies in treatment samples were compared to vehicle-treated samples to quantify the fold-change induced by each drug. **b** Unsupervised clustering demonstrated that TNBC cell response to cisplatin and PARPi strongly overlap, supporting the idea that these drugs elicit dependence on highly overlapping pathways. The heatmap shows the log_2_ fold change for each shRNA for the indicated drug treatment relative to vehicle-treated cells. Depleted shRNAs are indicated in blue, while overrepresented shRNAs are indicated in red. **c** Scatter plot visualization of shRNA depletion in cells treated with Chk1i; the log_2_ shRNA fold change (y-axis) is plotted against the log_10_ *p*-value (*x*-axis) for each gene. The analyses in **b** and **c** were performed using TIBCO Spotfire.

A synthetic-lethal drop-out screen in the TNBC cell line, BC3-A2^25^, was performed to identify gene products selectively required for survival in the presence of replication stress. The library was designed, by computational mining and manual curation, to target ~350 genes involved in cell cycle progression, DNA replication, and DNA damage repair (Supplementary Table 1). Since greater than 30% of these 350 genes are essential core fitness genes^26^, the feasibility of a CRISPR-based approach to detect synthetic lethal interactions were significantly reduced. Thus, we opted to use a pooled barcoded shRNA lentivirus library featuring ten shRNAs per gene to facilitate knockdown, but not a complete loss, of essential proteins like ATR, CHK1, and RPA2. Cells with shRNAs that target critical regulators of response to ATRi/Chk1i, would then be preferentially depleted from the cell population upon treatment with the pathway inhibitor. BC3-A2 cells were lentivirally transduced with the pooled library at a low MOI to achieve 1 integrant per cell, with a coverage of 700–1000 cells for each shRNA, and treated with puromycin to select a population of stably transduced cells (Fig. 1a). Next, transduced cells were exposed to a sub-lethal dose of the Chk1 inhibitor, LY2606368, to induce replication stress^12^. The LY2606368 concentration was chosen to inhibit the kinase activity of Chk1, but not Chk2. As shown in Supplementary Fig. 1a (lane 1 vs. 2), exposure to cisplatin was sufficient to increase autophosphorylation of both Chk1 (S296) and Chk2 (S516). While cisplatin-induced Chk1 autophosphorylation was inhibited by LY2606368, Chk2 autophosphorylation was unchanged in these samples (lane 2 vs. lanes 5 and 6). In addition to interrogating how Chk1i-induced replication stress increased the reliance on distinct proteins, we sought to compare the relative requirement of these proteins when DNA fidelity was challenged by cisplatin and PARP1/2 inhibition. Thus, we performed parallel synthetic lethal screens in the presence of cisplatin, which induces DNA cross-links, or talazoparib, which, in addition to inhibiting the enzymatic activity of PARP1/2 and inducing DNA double-strand breaks, also increases replication fork speed^27,28^. Upon recovery from treatment, cells were subjected to next-generation sequencing of barcodes to quantify the frequency of each shRNA. By comparing the barcode frequency in samples that were treated with vehicle vs Chk1i (or PARPi/cisplatin), we determined which shRNAs were preferentially depleted in response to each treatment.

The significant and preferential depletion of shRNAs causing knockdown of proteins known to be essential for cell viability during Chk1i-induced replication stress (RRM1, RRM2, and RPA2), PARP1/2 inhibition (RBBP8/CtIP, polq, Rad51), and cisplatin-mediated DNA cross-links (FANCI, FANCD2) provided validation of the screen (Supplementary Table 2). Comparison of shRNA depletion among the three drugs revealed clusters of genes required for survival in the presence of all three treatments, and others that were drug-specific (Fig. 1b; Supplementary Table 3). The genes required for cell viability under all three treatment conditions converged on DNA strand elongation during replication, with strong depletion of multiple targets involved in DNA polymerase processivity (RFC2/4/5, PCNA). In addition to inducing a significant dependence on genes required for DNA polymerase processivity, Chk1i was uniquely associated with a striking reliance on DNA primase activity (Supplementary Table 3—prim2, Supplementary Table 4—prim1, Supplementary Fig. 1b, c).

### Disruption of the cytosolic iron–sulfur assembly (CIA) pathway sensitizes cancer cells to replication stress

**Fig. 2:**
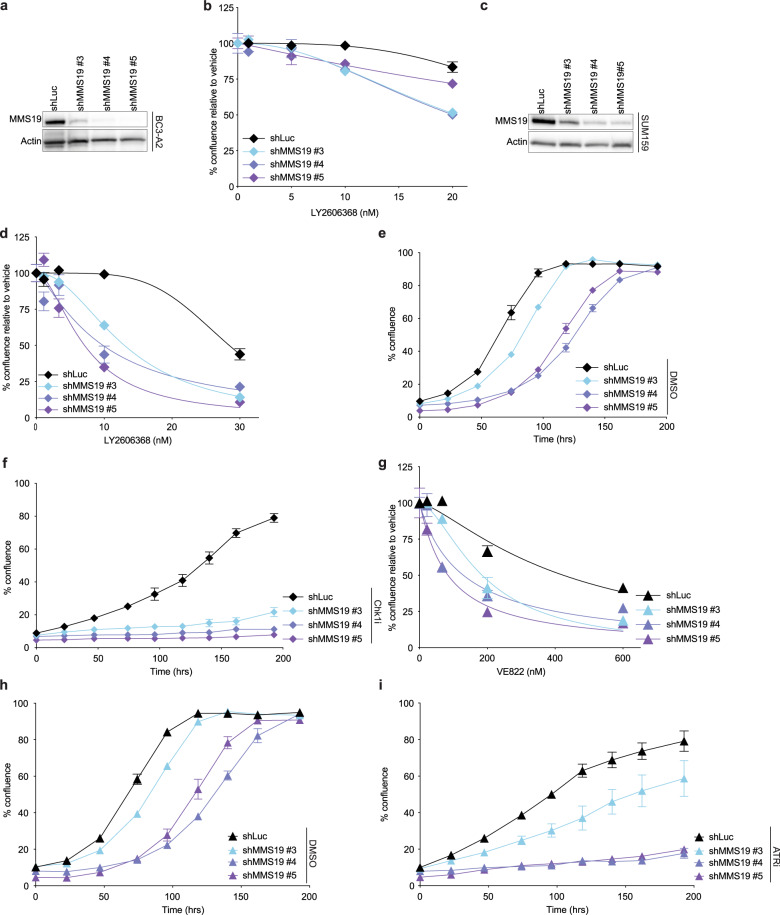
MMS19-depletion increases sensitivity to Chk1i and ATRi. **a** Western blot showing efficient shRNA-mediated depletion of MMS19 in BC3-A2 cells. **b** Control shLuc or MMS19-depleted BC3-A2 cells were plated at a density of 2.5 × 10^3^ cells per well in triplicate, allowed to attach overnight, and then treated for 120 h with the indicated doses of Chk1i. Cell growth was monitored by live-cell imaging; percent cell confluence is plotted (*y*-axis) against the concentration of Chk1i used (*x*-axis, LY2606368). **c** Western blot showing efficient shRNA-mediated depletion of MMS19 in SUM159 cells. **d** Control shLuc or MMS19-deficient SUM159 cells were treated and analyzed as described in panel b. **e**, **f** Control shLuc or MMS19-deficient SUM159 cells were treated for the indicated times with either DMSO or Chk1i (10 nM Ly2606368). Percent well confluence as a function of time is indicated. **g** Control shLuc or MMS19-depleted SUM159 cells were plated at a density of 2.5 × 10^3^ cells per well in triplicate and allowed to attach overnight, and then treated for 120 h with the indicated doses of ATRi. Cell growth was monitored by live-cell imaging; percent cell confluence is plotted (*y*-axis) against the concentration of Chk1i used (*x*-axis, LY2606368). **h**, **i** Control shLuc or MMS19-depleted SUM159 cells were treated for the indicated time periods with either DMSO or ATRi (200 nM VE-822). Percent well confluence as a function of time is indicated. **b**, **d**–**i** Data point—mean; error bars—SEM of three independent experiments for all graphs except for (**e**) and (**f**) (two independent experiments).

**Fig. 3:**
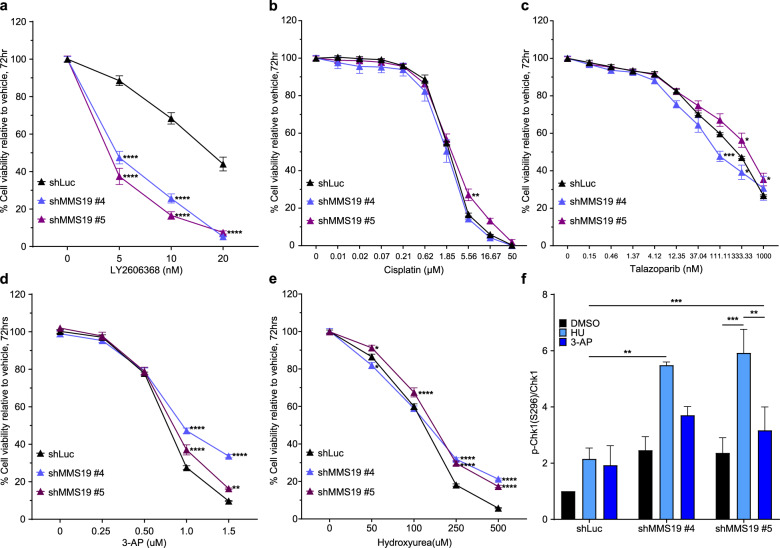
Unique sensitization of MMS19 depleted SUM159 cells to Chk1i. **a**–**e** SUM159 cells were seeded at a density of 2.5 × 10^3^ cells per well in 96-well, clear-bottom tissue culture plates in 100 µL complete growth medium, and then incubated overnight at 37 °C in 5% CO_2_. Cells were then treated in triplicate with the indicated concentrations of drugs or with vehicle (saline for cisplatin and DMSO for all other treatments). Endpoint assays were performed at 72 h post treatment. Cell viability measurements from drug-treated samples were normalized to their respective vehicle-treated controls; Data point—mean; error bars—SEM of three or more independent experiments, each done in triplicates. **f** SUM159 cells were stably transduced with a non-targeting shRNA (shLuc) or two different shRNAs targeting MMS19. Cells were treated for 20 h with DMSO, 1 mM hydroxyurea, or 0.5 µM 3-AP. Total cell lysates were analyzed for phospho-Chk1 S296 (autophosphorylation site) and total Chk1 by Western blotting. The ratio of p296-Chk1 to total Chk1 was calculated and graphed as mean; error bars—SEM of three independent experiments. *p < 0.05, **p < 0.01, and ***p < 0.001 ****p < 0.0001 (two-way ANOVA, Turkey’s multiple comparisons test).

The drop-out screen also revealed significant depletion of MMS19-targeting shRNAs in cells treated with Chk1i (Fig. 1c), but not cisplatin or PARPi (Supplementary Table 3), which suggested a preferential requirement for the MMS19 protein in the ATR/Chk1-mediated RSR. Given that MMS19 was among the top 10% of hits in Chk1i treated cells and, importantly, that it conferred a dependency that was unique to Chk1i, we chose to further explore the role of MMS19 in regulating cell response to perturbations in the ATR/Chk1 pathway. MMS19 has a central role in the transfer of Fe–S clusters to recipient apoproteins in the CIA pathway, which facilitates the maturation and proper functioning of many proteins^29,30^. To validate our screen results and elucidate how CIA disruption affects the response to Chk1i, we transduced BC3-A2 cells with lentivirus encoding different shRNAs against MMS19, and puromycin selected to generate a stably transduced population of cells (Fig. 2a). MMS19-depleted cells were treated with increasing concentrations of Chk1i and cell proliferation was evaluated with live-cell imaging to track cell growth. Consistent with the screen results, loss of MMS19 was associated with increased sensitivity to Chk1i (Fig. 2b). These results were confirmed in two additional TNBC cell lines, SUM159 (Fig. 2c, d, Supplementary Fig. 2) and MDA-MB-231 (Supplementary Fig. 3). Analysis of cell growth over time revealed that although depletion of MMS19 by itself was associated with decreased cell proliferation (Fig. 2e), treatment with Chk1i resulted in a growth inhibition greater than this effect (Fig. 2f). Although MMS19-depletion sensitized cells to Chk1i, its depletion did not sensitize cells to cisplatin or to PARPi (Fig. 3), confirming our screen results. In addition, the exquisite sensitivity of MMS19-depleted cells to Chk1i was not observed when cells were treated with either triapine (3-AP) or hydroxyurea, agents that deplete nucleotides by inhibiting ribonucleotide reductase (Fig. 3). These results highlight the unique sensitivity of MMS19-depleted cells to deregulation of origin firing, which is specific to inhibition of the ATR/Chk1 pathway.

Next, Chk1 kinase activity was monitored to determine the impact of MMS19 depletion on activation of the Chk1 pathway. Chk1 kinase activity can be assessed indirectly by monitoring the ability of Chk1 to autophosphorylate on serine 296 (pS296). In DMSO-treated samples, levels of pS296-Chk1 were elevated in MMS19-depleted cells relative to shLuc-control cells although the difference did not reach statistical significance (Fig. 3f). Hydroxyurea treatment (and to a lesser extent 3-AP treatment) resulted in a significant increase in pS296-Chk1 in MMS19-depleted cells relative to shLuc-control cells. These findings support the screen results indicating that MMS19-deficiency creates a dependency on the Chk1 pathway for cell survival. Consistent with enhanced sensitivity to Chk1i, MMS19-depleted cells also exhibited increased sensitivity to ATR inhibition (Fig. 2g–i).

**Fig. 4:**
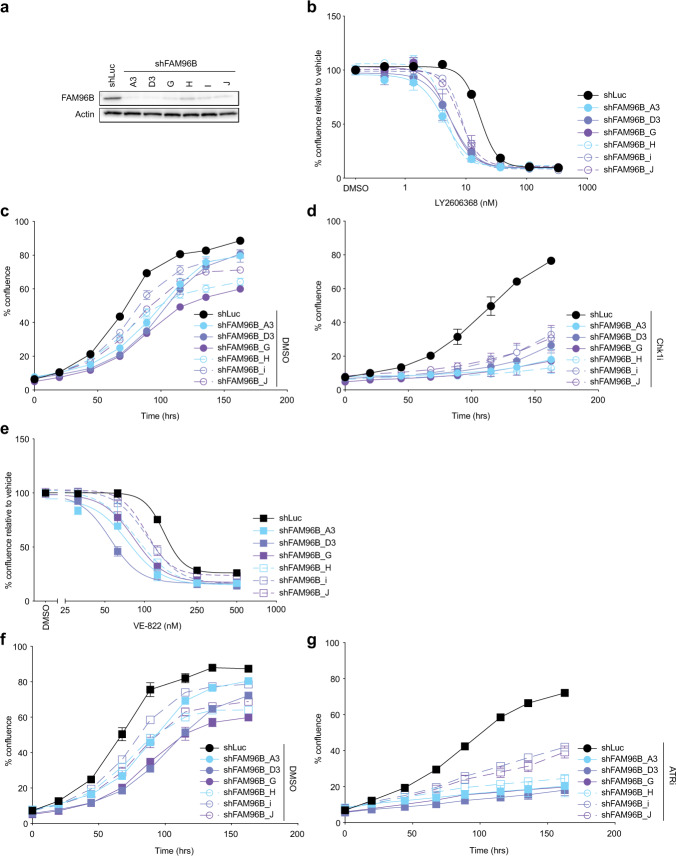
Loss of CIA2B-FAM96B increases sensitivity to inhibition of the ATR/Chk1 pathway. SUM159 cells were stably transduced with a non-targeting shRNA (shLuc) or 6 different shRNAs targeting CIA2B-FAM96B. **a** CIA2B-FAM96B knockdown was confirmed by Western blotting. **b** Control shLuc or FAM96B-depleted SUM159 cells plated at a density of 2.5 × 10^3^ cells per well in triplicate were allowed to attach overnight, and then treated for 135 h with the indicated doses of Chk1i. Cell growth was monitored by live-cell imaging; percent cell confluence is plotted (*y*-axis) against the concentration of Chk1i used (*x*-axis, LY2606368). **c**, **d** Control shLuc or FAM96B-depleted SUM159 cells were treated for 135 h with either DMSO or Chk1i (10 nM Ly2606368). Percent well confluence as a function of time is indicated. **e** Control shLuc or FAM96B-deficient SUM159 cells plated at a density of 2.5 × 10^3^ cells per well in triplicate were allowed to attach overnight, and then treated for 135 h with the indicated doses of ATRi (VE-822). Cell growth was monitored by live-cell imaging; percent cell confluence relative to DMSO treated cells is plotted (*y*-axis) against the concentration of ATRi used (*x*-axis, VE-822). **f**, **g** Control shLuc or FAM96B-depleted SUM159 cells were treated for 135 h with either DMSO or ATRi (200 nM VE-822). Percent well confluence as a function of time is indicated. **b**–**g** Data point—mean; error bars—SEM of three independent experiments.

To further examine the role of the CIA pathway in cancer cell response to replication stress, we used shRNAs to determine how the loss of CIA2B-FAM96B, a CIA protein that was not targeted in the original library, affected sensitivity to Chk1i and ATRi. CIA2B-FAM96B was chosen because it forms a complex with MMS19 to regulate cytosolic iron-sulfur protein assembly. In alignment with MMS19-deficiency, CIA2B-FAM96B knockdown also increased sensitivity to Chk1i (Fig. 4a–d) and ATRi (Fig. 4e–g).

**Fig. 5:**
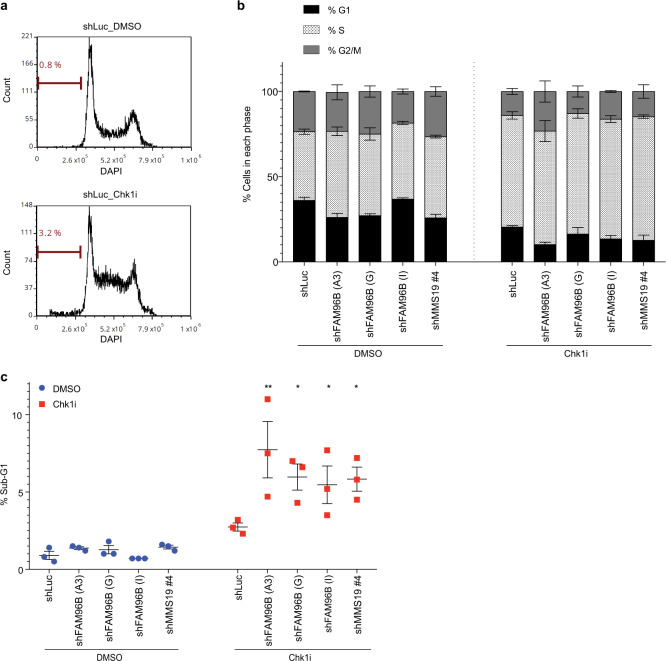
Chk1i induces expansion of the fraction cells in S-phase in control and MMS19- or CIA2B-FAM96B-depleted cells. shLuc, shMMS19, or shCIA2B-FAM96B cells were treated with 10 nM Chk1i for 24 h prior to being processed by flow cytometry. Cells were fixed with 70% ethanol then DAPI stained to allow for DNA quantification. **a** Representative histograms of shLuc cells demonstrates increased S-phase fraction along with modest increase in sub-G1 apoptotic fraction in Chk1i vs. DMSO-treated cells. DNA content analyses were performed using the ModFit module in fcsExpress software. **b** The fraction of cells in G1, S, and G2/M was quantitated using FCS Express. Datapoint—mean; error bars—SEM of three independent experiments. **c** Quantification of the fraction of sub-G1 cells for each sample reveals statistically significant higher levels in sub-G1 population in Chk1i-treated shCIA2B-FAM96B- and shMMS19-depleted cells compared to shLuc cells. Datapoint—mean; error bars—SEM of three independent experiments. *p < 0.05, **p < 0.005 (two-way ANOVA, Dunnett’s multiple comparisons test).

To determine if increased sensitivity to Chk1i was associated with differential effects of Chk1i on cell cycle progression of control vs. MMS19- or CIA2B-FAM96B-depleted cells, flow cytometry was used to evaluate cell cycle profiles. Cells were treated with Chk1i or DMSO for 24 h, then subjected to ethanol fixation and DAPI staining to determine DNA content. Exposure of control shLuc cells to Chk1i increased the fraction of cells in S-phase, and was also associated with a modest increase in the apoptotic sub-G1 fraction (Fig. 5a, c, Supplementary Fig. 4). Compared to shLuc cells, no significant difference in the overall cell cycle distribution was observed in DMSO-treated CIA2B-FAM96B- or MMS19-depleted cells; the addition of Chk1i to these cells was not associated with a statistically significant difference in cell cycle distribution (Fig. 5b). However, these cells featured higher levels of sub-G1 apoptotic fraction, compared to shLuc control cells upon addition of Chk1i (Fig. 5c), in alignment with our observations that loss of CIA2B-FAM96B or MMS19 increased sensitivity to Chk1i.

### Disrupting the CIA through loss of MMS19 or CIA2B-FAM96B alters the pattern of metabolites

RNAi-mediated depletion of MMS19 and CIA2B-FAM96B has been reported to affect Fe–S maturation of proteins involved in nucleotide metabolisms, such as GPAT and DPYD^30,31^. Alterations in the levels or ratios of nucleotides can disrupt cell energetics and genomic instability, and cells have evolved a complex feedback network to carefully regulate the homeostasis of nucleotides and their derivative metabolites^32–35^. In alignment with previous reports, both shCIA2B-FAM96B and shMMS19 cells featured decreased DPYD protein levels (Supplementary Fig. 5), which was not affected by Chk1i. Given the central roles for GPAT and DPYD in catalyzing the rate-limiting steps of purine biosynthesis and pyrimidine catabolism respectively, we hypothesized that loss of CIA2B-FAM96B or MMS19 would impact nucleotide homeostasis. To test this hypothesis, we used mass spectrometry to comprehensively measure the levels of nucleotides and their derivative metabolites in SUM159 cells depleted for either shMMS19 or shCIA2B-FAM96B after exposure to DMSO or Chk1i. Under DMSO treated conditions, both shMMS19 and shCIA2B-FAM96B-depleted cells featured altered patterns of metabolite concentrations relative to control shLuc cells (Supplementary Fig. 6), with statistically significant changes in several important metabolites such as IMP and UMP, central intermediates in de novo purine and pyrimidine synthesis. While Chk1i-treated shLuc cells featured reduced levels of most metabolites, this was not observed in Chk1i-treated shCIA2B-FAM96B-depleted cells (Supplementary Fig. 6). Interestingly, Chk1i-treated shMMS19-depleted cells had an intermediate phenotype between Chk1i-treated shLuc and shCIA2B-FAM96B-depleted cells. The overall changes in levels of purine and pyrimidine metabolites suggest deregulation of nucleotide homeostasis, which can affect the fidelity of RNA and DNA synthesis and cause decreased replication stress tolerance.

## Discussion

Here, we report that cells deficient in either MMS19 or CIA2B-FAM96B, components of the cytosolic iron–sulfur (Fe–S) protein assembly (CIA) complex, exhibit a unique sensitivity to Chk1 and ATR inhibitors relative to cisplatin, talazoparib (PARPi), hydroxyurea and 3-AP. MMS19 together with CIA2B-FAM96B and C1A1 form the CIA targeting complex that facilitates Fe-S cluster insertion into most cytosolic and nuclear apoproteins. Many of the CIA-client proteins are nuclear proteins with roles in DNA replication and repair, including DNA polymerases α, δ, ε, and ζ, and DNA helicases XPD, RTEL1, FANCJ, DNA2, and DDX11. Loss of the Fe–S cluster often leads to reduced protein function and disease-causing mutations of XPD, FANCJ, and MUTYH has been mapped to their Fe–S domains^29,30,33,36^. Given the dependency of critical DNA replication and repair proteins on Fe–S co-factors, we asked if a deficiency in components of the CIA-targeting complex would sensitize TNBC to inhibitors that induce replication stress.

Each inhibitor used in our screen is known to induce replication stress either by (i) affecting the fidelity of the DNA template through inhibition of DNA damage repair (PARPi), (ii) creating lesions that affect replication fork progression (PARP1i, cisplatin), or (iii) destabilizing fork equilibrium and disrupting its supply of raw material by deregulating origin firing (Chk1i). Accordingly, all three agents induced dependency on proteins involved in DNA synthesis, including the DNA polymerase accessory factors RFC2 and RFC4, and the processivity factor PCNA. Chk1i was characterized by a striking and unique dependence on specific components of the fork, including primase-2, POLD1, and POLE, all of which are client proteins of MMS19^36^. While both cisplatin and PARPi are expected to cause disruption of active replication forks, loss of Chk1 activity further disrupts replication fork equilibrium by aberrantly increasing dormant origin firing, which increases the density of replication forks in a given nucleus. The increase in fork density is expected to heighten the demand for primase activity at the replication bubble and put the cell at risk for primase exhaustion and replication catastrophe. This dependence on DNA primase activity highlights the critical role of Chk1 in ensuring strategic timing of origin firing to avoid exhaustion of critical fork components.

We demonstrated that inhibition of the ATR/Chk1 pathway induces selective dependency on the CIA pathway. Chk1 and ATR inhibitors do not directly damage DNA, but instead impede repair of endogenously induced damage, and deregulate cell cycle checkpoints and replication forks. The link between components of the CIA pathway and DNA maintenance was initially reported in *Saccharomyces cerevisiae*, where homozygous MMS19 mutants showed increased sensitivity to MMS-induced DNA alkylation and UV damage, but not X-ray irradiation^37^. Additional studies probed the mechanism of heightened sensitivity to UV damage and revealed that MMS19 associates with a critical component of the NER pathway, XPD, which was later shown to require Fe–S cluster insertion for its helicase activity^38,39^. Here, we show that disrupting MMS19 or CIA2B-FAM96B impacts cell viability in the absence of exogenous damage, by reducing tolerance to replication stress that occurs upon abrogation of the ATR/Chk1 pathway.

We also demonstrated that loss of either MMS19 or CIA2B-FAM96B is sufficient to alter the homeostasis of nucleotides and derivative metabolites in cancer cells, which likely contributes to the observed decrease in replication stress tolerance. Nucleotide homeostasis is controlled by an intricate feedback system that balances de novo synthesis with salvage and degradation pathways. The complexity of the nucleotide regulatory network presents a considerable challenge to pinpointing the exact mechanisms by which CIA disruption alters nucleotide homeostasis, and extensive biochemical studies are required to address this challenge. Nevertheless, there is substantial evidence that deregulating nucleotide homeostasis impacts the fidelity of RNA and DNA synthesis and decreases genomic stability^32,35,40,41^. A connection between CIA-disruption and compromised genomic stability was elegantly shown in a 2018 study by Weon et al. ^42^, which demonstrated that post-translational regulation of MMS19 via E3 ubiquitin ligase mediated degradation is enhanced in a number of cancers including lung, ovarian, and head and neck cancers. Strikingly, the authors found that amplification of MAGE-F1, a component of the E3 ubiquitin ligase complex that regulates MMS19 degradation, was associated with significant increases in total mutation burden in lung and head and neck squamous cell carcinomas. MAGE-F1 amplification was further associated with a significant reduction in the overall survival of patients with head and neck cancers. Intriguingly, the genes encoding MMS19 and CIA2B-FAM96B both reside in close proximity to tumor suppressor genes (PTEN and CDH1 respectively) that are frequently deleted in cancer, which increases susceptibility to passenger deletion. In fact, our analysis of TCGA data revealed that many cancers feature heterozygous deletion of MMS19 or CIA2B-FAM96B (Supplementary Table 5)^43,44^, and provides another compelling link between tumorigenesis and the fidelity of the CIA pathway.

In this study, we demonstrated that depletion of components of the CIA-targeting complex sensitizes TNBC cells to Chk1 and ATR inhibitors. Future studies are aimed at providing a more detailed mechanistic understanding by identifying the proteins that underlie Chk1i/ATRi dependency in the context MMS19 and CIA2B-FAM96B deficiency and determining the role of Fe–S clusters in these proteins. Given the emerging role of the CIA pathway in genomic stability and cancer progression, and the selective sensitivity that CIA disruption confers to cancer therapies, understanding the mechanistic nuances will be critical for informing efficacious therapeutic intervention.

## Methods

### Cell culture and lentiviral transduction

BC3-A2 (Washington University, St Louis, MO), SUM159 (MD Anderson Cancer Center, Houston, TX), and MDA-MB-231 cells (ATCC, Cat # CRM-HTB-26), were cultured in DMEM supplemented with 10% FBS. Cell line authenticity was confirmed by STR profiling, and all cells were tested for mycoplasma contamination. For lentiviral transduction, 293 T cells were transfected (TransIT^®^-LT1 transfection reagent, Mirus #MIR 2304) with target constructs along with packaging (pCMV delta R8.2) and envelope (pCMV-VSV-G) plasmids. Virus-containing media was passed through 0.45-micron filters and used to transduce target cells at a multiplicity of infection (MOI) of 0.3, followed by puromycin- selection of transduced cells. Drug treatments were performed 5–7 days post puromycin selection.

### shRNAs

shRNAs were in the pLKO.1 vector, with sequences and TRC number as follows:

*Primase-2*: PRIM2_#2: TRCN0000000200 (CTACCCTCATTGCCTTCAGTT); PRIM2_#3: TRCN0000000201 (CACGAAGAAGAGATCATATTT).

*MMS19*: MMS19_#3: TRCN0000107927 (CCTGCCTCGAAATGTGGAAAT); MMS19_#4: TRCN0000107928 (CCGACACACAGTCTACAATAT); MMS19_#5: TRCN0000107929 (TGGAGCTATGAAGACAAAGAT);

*CIA2B-FAM96B*: CIA2B-FAM96B_A3: TRCN0000134659 (GAACAAGCAACTTGCAGATAA); CIA2B-FAM96B_D3: TRCN0000275705 (GAACAAGCAACTTGCAGATAA); CIA2B-FAM96B_G: TRCN0000135153 (CTAGAGGAGTTGAACGTAGTA); CIA2B-FAM96B_H: TRCN0000138077 (GCAGTGAACAAGCAACTTGCA); CIA2B-FAM96B_I: TRCN0000137561 (CGCGAGATCTTCGATCTGATT); CIA2B-FAM96B_J: TRCN0000282035 (CTAGAGGAGTTGAACGTAGTA).

### shRNA library design and construction

A customized barcoded shRNA library was designed to target ~350 genes, with a coverage of 10 shRNAs/gene, constructed in the pRSI16 vector (Cellecta) and used to generate high titer lentivirus (>2 × 10^8^ TU).

### Western blotting and antibodies

Standard Western blotting procedures were used. Briefly, cultured cells were washed with 1X PBS then lysed with RIPA buffer supplemented with protease and phosphatase inhibitors (Sigma catalog number 5892791001 and 4906837001, respectively). Lysates were heat-denatured prior to loading onto 4–20% gels (Bio-Rad catalog number 567–1094) and detected with antibodies as detailed next. Proteintech: MMS19 (16015-1-AP, dilution 1/1000), FAM96B (20108-1-AP, dilution 1/500); ThermoFisher Scientific: DPYD Monoclonal Antibody (7D4) (H00001806-M01, dilution 1/1000); Cell Signaling: phospho-Chk2 S516 (2669S, dilution 1/1000), Chk1 (2360 S, dilution 1/1000), GAPDH (8884 S, dilution 1/1000), β-Actin (12620, dilution 1/2000); Abcam: phospho-Chk1 S296 (ab79758, dilution 1/500). All blots derive from the same experiment and were processed in parallel. Uncropped and unprocessed scans of western blots can be found in the Supplementary Information.

### IncuCyte live-cell imaging

Cells were seeded at 2.5 × 10^3^ cells per well in 96-well, clear-bottom tissue culture plates in 100 µL complete growth medium, and inserted into the IncuCyte ZOOM (Essen Bioscience, Ann Arbor, MI, USA), which was housed in a humidified incubator at 37 °C, 5% CO_2_ with four fields imaged per well under 10× magnification. The next day, cells were treated with a vehicle or drug, and plates were imaged every 8 h for a total of three days. Images were analyzed using IncuCyte ZOOM software. All assays were performed in triplicate.

### CellTiter-Glo(CTG) luminescent cell viability assay

Cells were seeded at a density of 2.5 × 10^3^ cells per well in 96-well, clear-bottom tissue culture plates in 100 µL complete growth medium and incubated at 37 °C in 5% CO_2_. The next day, cells were treated in triplicate with drugs or vehicles. Cell viability was assayed 72 h post-treatment using CellTiter-Glo luminescent cell viability assay (Promega, Cat# G7572, Madison, WI). Briefly, at the endpoint, plates were equilibrated to room temperature for 30 min, after which 100 µL CellTiter-Glo assay solution was added to each well according to the manufacturer’s recommended 1:1 vol/vol ratio. Following incubation for 10 min with shaking at room temperature, cell-CTG solution mixtures of each well were transferred to a 96-well flat-bottom (chimney) plate (Cat# 655073, Greiner Bio-One, Frickenhausen, Germany), and then measured for luminescence using CLARIOstar plate reader (BMG Labtech, Ortenberg, Germany). Cell viability measurements were normalized to their respective vehicle-treated controls. All CTG assays were performed in triplicate.

### Flow cytometry

SUM159 cells were seeded at 1.5 × 10^6^ cells per 10 cm tissue culture dish. The following day, cells were treated with DMS0 or 10 nM LY2606368. Cells were collected 24 h later, stained with a live/dead viability dye (Ghost dye red 780, Fisher 501052988) then fixed with 70% ice-cold ethanol. Cells were washed in PBS, permeabilized with 0.5% Triton X-100 in PBS, and DNA was stained with 10 µg/mL DAPI (Fisher #26-829-810MG) for determining DNA content. Data were collected using a Beckman Coulter Gallios, three laser configurations.

### Quantification of dNTPS

SUM159 cells were transduced with control shRNA (shLuc) or shRNAs specific for either FAM96B or MMS19 followed by puromycin selection. Five days later cells were suspended in 1 mL methanol and 0.25 mL water and lysed on a MM400 mill mixer at 25 Hz for 1 min × 2 with the aid of two 5-mm stainless steel balls, followed by sonication for 3 min in an ice–water bath. The tubes were clarified at 21,000 × *g* in an Eppendorf 5420R centrifuge and at 4 °C for 15 min. Clear supernatants were collected for assay of nucleotides using a Dionex 3400 UHPLC system coupled to a 4000 QTRAP triple-quad mass spectrometer (MS). The MS instrument was operated in the multiple reaction monitoring (MRM) mode with negative-ion (−) detection.

A 500 μL of each supernatant was precisely transferred to another Eppendorf tube and was added with 100 μL of water containing 13C10-GTP (internal standard) and 300 μL of chloroform. The mixture was vortex-mixed for 1 min and then centrifuged at 21,000 × *g* and 10 °C for 6 min. Totally, 300 μL of the upper aqueous phase was precisely taken out and dried in a speed-vac concentrator. The residue was reconstituted in 300 μL of 20% acetonitrile. A 10 μL was injected for quantitation of nucleotides by ion-pairing HPLC–(−)ESI–MRM/MS using a BEH C18 (2.1 × 150 mm, 1.8 μm) UPLC column. The mobile phase was composed of (A) 5 mM tributylamine (pH 7) in water and (B) acetonitrile as the mobile phase for gradient elution. The flow rate was 0.25 mL/min and the column temperature was 50 °C.

An STD mix containing standard substances of all the targeted nucleotides was dissolved in 20% acetonitrile containing 13C10-GTP as internal standard. This standard solution (S1) was serially diluted in a dilution ratio of 1–4 with the same solvent to prepare standard solutions S2–S8. A 10 μL of each standard solution was injected to record the data files so as to generate linear-regression calibration curves with internal calibration. Concentrations of detected nucleotides in individual samples were calculated by interpolating the calibration curves with the measured analyte-to-internal standard peak area ratios. Sciex MultiQuant was used for data processing. Experiments were performed in triplicate.

### Obtaining biological materials

All unique materials are available from the authors upon request or from commercial sources as described in the manuscript.

### Reporting summary

Further information on research design is available in the Nature Research Reporting Summary linked to this article.

## Supplementary information

Supplemental Material

Reporting Summary

## Data Availability

The data supporting the findings of this study are available within the paper and its Supplementary Information files.

## Code availability

No unique code or algorithms were used for the collection or analysis of data contained in this paper.
