# Supplementary material for: The cytosolic iron–sulfur cluster assembly (CIA) pathway is required for replication stress tolerance of cancer cells to Chk1 and ATR inhibitors
Source: NPJ Breast Cancer. 2021 Dec 2;7:152. doi: 10.1038/s41523-021-00353-2 (PMC8639742; doi:10.1038/s41523-021-00353-2)

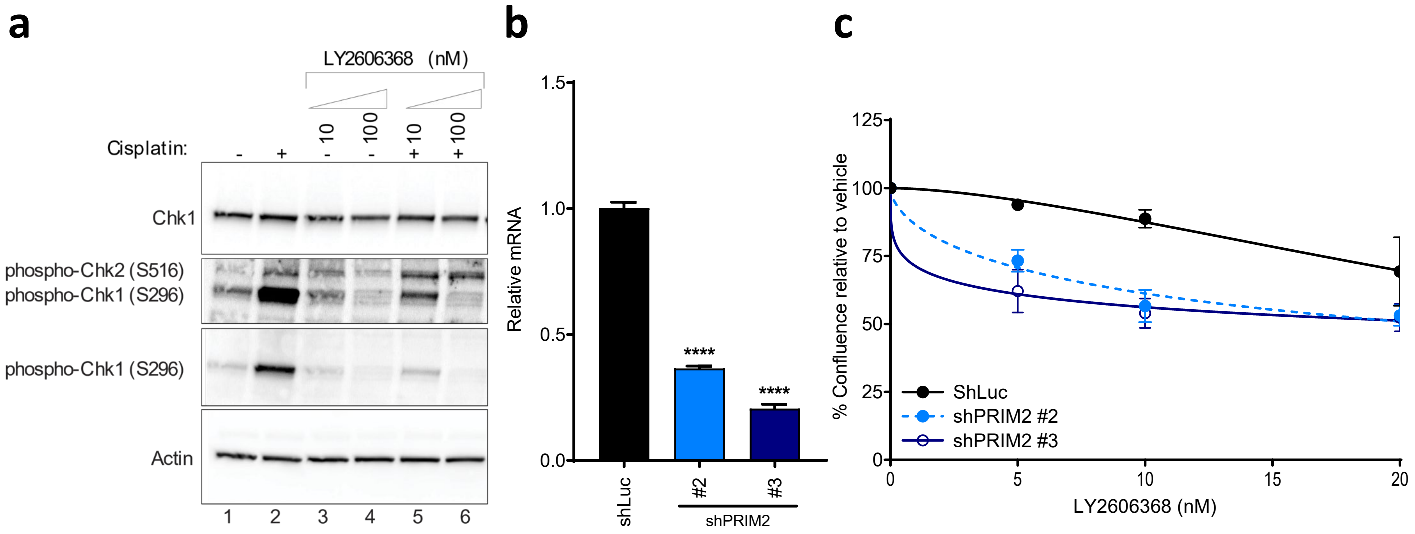

Supplementary Figure 1. a LY2606368 efficiently inhibits Chk1 but not Chk2 in BC3-A2 cells. Cells were treated with 5 μM cisplatin (or saline) for 16h to induce the DNA damage response pathway and increase activation of Chk1 and Chk2. To confirm efficient Chk1 inhibition by LY2606368, cells were treated with 10 or 100 nM LY2606368 for 3 h, and Chk1 and Chk2 phosphorylation was evaluated by Western blotting. **b, c** Depletion of primase-2 increases sensitivity of BC3-A2 cells to Chk1i. BC3-A2 cells were transduced with a non-targeting shRNA (shLuc) or with 2 different primase-2 targeting shRNAs at an MOI of 0.3. The following day, cells were cultured in the presence of puromycin. Five days later, control shLuc and primase-2-deficient BC3-A2 cells were subjected to qPCR to confirm knockdown (b) or were plated at a density of 2.5 x 10^3 cells per well in triplicate and allowed to attach overnight, and then treated for 120 h with various doses of LY2606368 (c). Statistical analysis in b was performed using ordinary one-way ANOVA with multiple comparisons; n=9 biological replicates; bar – mean; error bars – SEM; ****$p < 0.0001$ In panel c, cell growth was monitored by live cell imaging; percent cell confluence wad plotted (y-axis) against the concentration of Chk1i used (x-axis, LY2606368). Data point – mean; n=3 biological replicates; error bars – SEM.

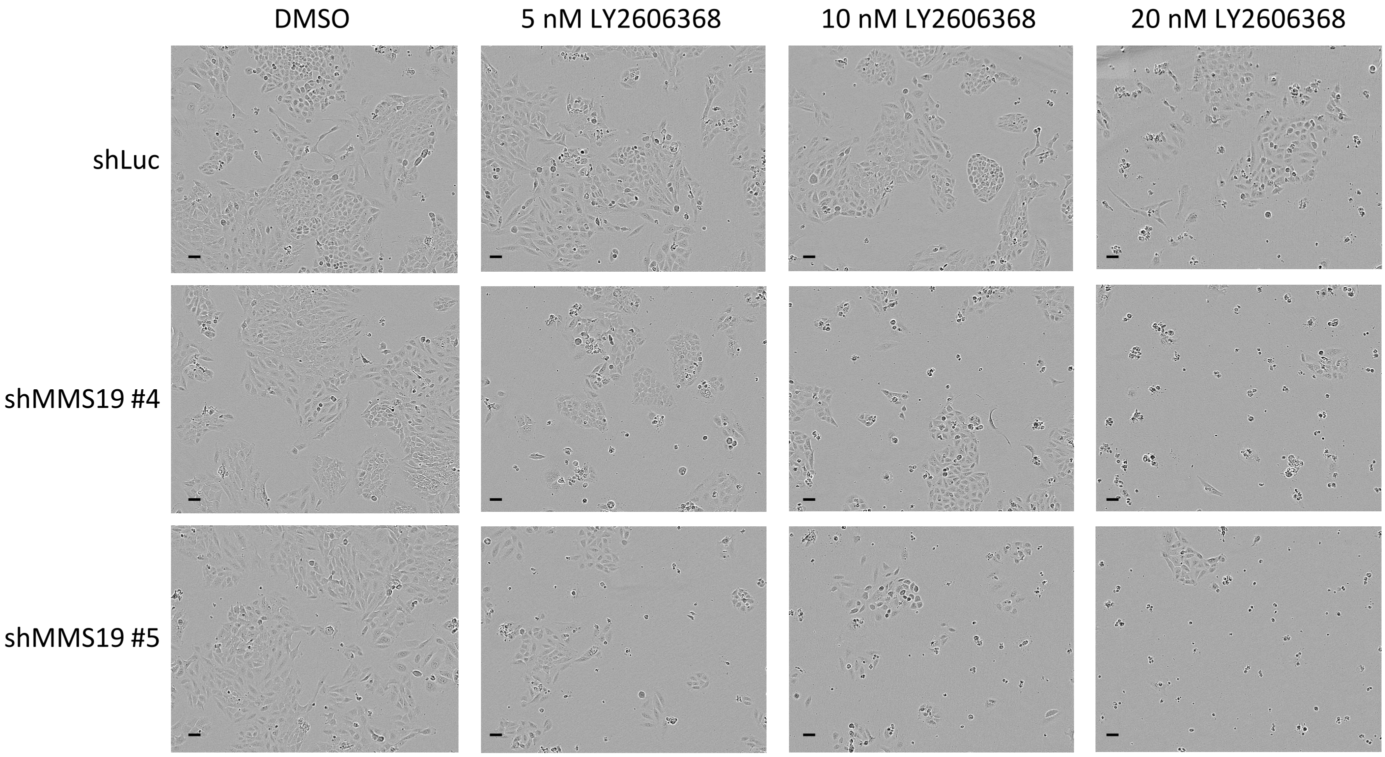

Supplementary Figure 2. Dose-dependent loss of cell viability in MMS19-deficient SUM159 cells treated with Chk1i. SUM159 cells were stably transduced with a non-targeting shRNA (shLuc) or two different MMS19-shRNAs. Cells were treated for 72 h with DMSO or the indicated dose of LY2606368. Representative phase contrast images of four fields taken per well are shown. Scale bar = 50 uM at 10 x magnification. Images were acquired using the IncuCyte ZOOM Live-Cell imaging Instrument (Essen Bioscience, Ann Arbor, MI, U.S.A.)

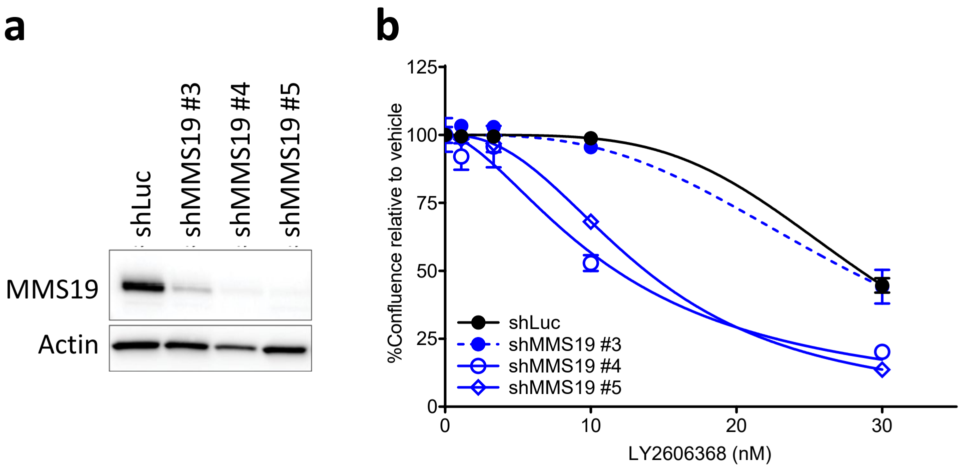

Supplementary Figure 3. MMS19 depletion sensitizes MDA-MB-231 cells to Chk1i. **a** Western blot showing efficient shRNA-mediated depletion of MMS19 in MDA-MB-231 cells. **b** Control shLuc or MMS19-deficient MDA-MB-231 cells were plated at a density of 2.5 x 10^3 cells per well in triplicate, allowed to attach overnight, and then treated for 235 h with the indicated doses of Chk1i. Cell growth was monitored by live cell imaging; percent cell confluence is plotted (y-axis) against the concentration of Chk1i used (x-axis, LY2606368). Data point – mean; error bars – SEM.

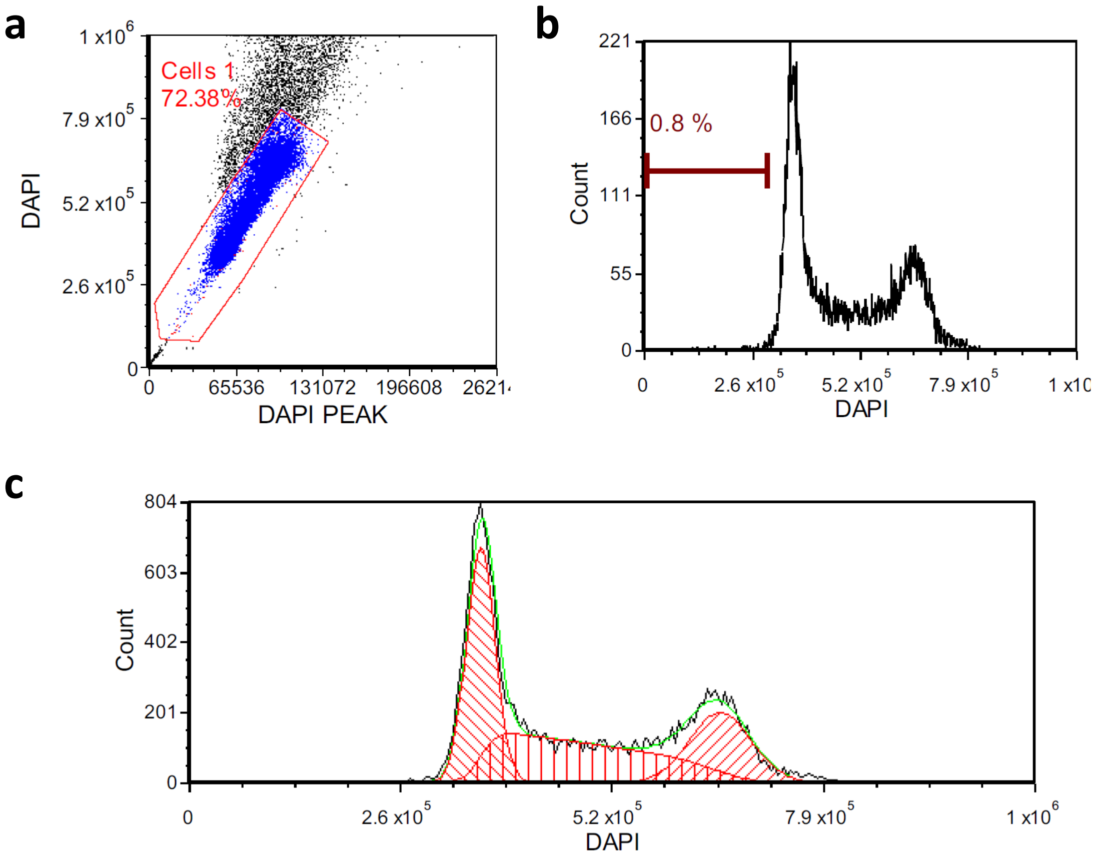

Cycle	G1 Mean	G1 CV	%G1	G2 Mean	G2 CV	%G2	%S	G2/G1	%Total	B.A.D.
Diploid	362662.30	4.98	37.08	660915.81	5.89	23.81	39.12	1.82	100.00	0.78

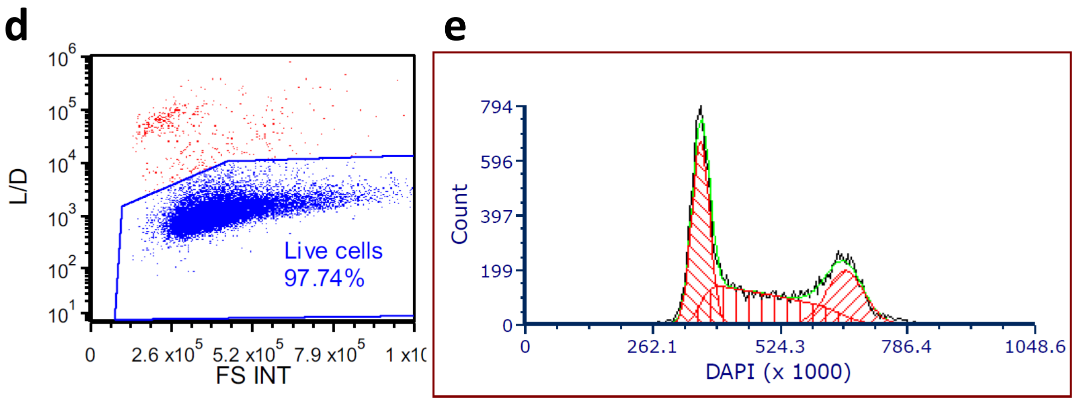

Cycle	G1 Mean	G1 CV	%G1	G2 Mean	G2 CV	%G2	%S	G2/G1	%Total	B.A.D.
Diploid	362705.31	4.96	37.17	661183.69	5.85	23.52	39.31	1.82	100.00	0.73

Supplementary Figure 4. Gating strategy used for data shown in Figure 5. Cells were stained with LIVE/DEAD™ Fixable Near-IR Dead Cell Stain Kit per manufacturer's instructions, washed and then fixed with 70% ethanol before adding DAPI to label DNA per standard protocol. **a** A single cell gate based on proportional DAPI-H vs DAPI-A signals was applied to exclude aggregated cell events by raw pulse shape. **b, c** Single cells were used for DNA content analysis to identify percentage of sub-G1, G1, S, and G2 cells. **d** From the singlet population, Live/Dead (L/D) Fixable positive dead cells were excluded. **e** Resulting live, single cells were used for DNA content analysis. All DNA content analysis performed using the ModFit module in fcsExpress software.

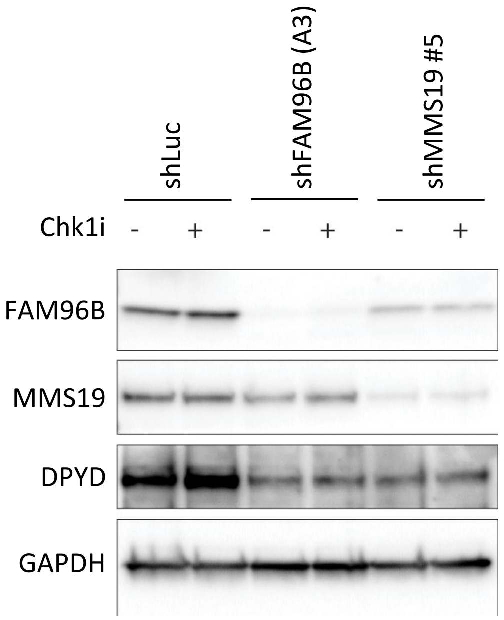

Supplementary Figure 5. Reduced levels of DPYD in SUM159 cells depleted for MMS19 or FAM96B. SUM159 cells were stably transduced with a non-targeting shRNA (shLuc) or shRNAs specific for either MMS19 or FAM96B. Cells were treated for 2 h with either DMSO (-) or 30 nM LY2606368 (+). The indicated proteins were analyzed by Western blotting.

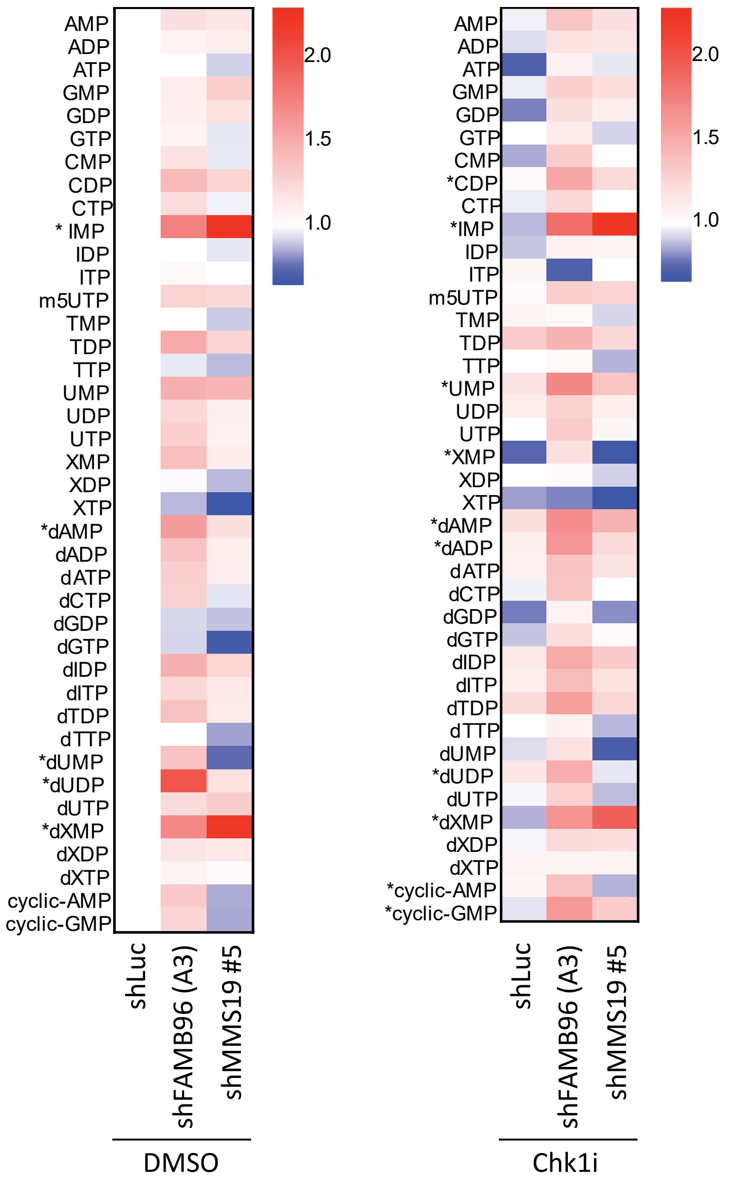

Supplementary Figure 6. Disrupting the CIA pathway alters the equilibrium of nucleotide production. Control, CIA2B-FAM96B- or MMS19-deficient SUM159 cells were treated with DMSO or Chk1i for 2 h and then subjected to mass spectrometry to quantify levels of nucleotides and their intermediate metabolites. The quantity of metabolite for each sample was normalized to DMSO-treated control (shLuc) cells and used to generate the heat maps, which represent the mean from three biologically independent experiments. Heatmap colors indicate changes in metabolites relative to control shLuc cells (white), decreased and increased levels of metabolites are shown in blue and red respectively. The heatmaps were generated using GraphPad Prism software. Statistical analysis using the two-way repeated measures ANOVA with multiple comparisons shows that a number of metabolites have statistically significant different levels (asterisks) compared with the shLuc cells, both at baseline and after Chk1i treatment, p values are given in the supplementary table 6.

Supplementary Table 1. DDRome Genes

gene_id

ABCF2	CDK7	FANCB	MLH3	POLG	RFC4	TFF2	ZW10
ABL1	CDKN1A	FANCC	MMS19	POLG2	RFC5	TGFB1	ZWINT
AIFM1	CDKN2A	FANCD2	MNAT1	POLH	RFWD2	TIMELESS	
ALKBH1	CDKN2B	FANCE	MPG	POLI	RINT1	TIPIN	
ANKRD17	CDKN2D	FANCF	MRE11A	POLK	RNF168	TNFRSF10B	
ANTXR1	CDT1	FANCG	MSH2	POLL	RNF8	TNP1	
APAF1	CEBPG	FANCI	MSH3	POLM	RPA1	TOP1	
APEX1	CENPF	FANCL	MSH4	POLN	RPA2	TOP2A	
APEX2	CETN2	FANCM	MSH5	POLQ	RPA3	TOPBP1	
APTX	CHAF1A	FEN1	MSH6	POLR2G	RPA4	TP53	
ASF1A	CHAF1B	FOXN3	MTOR	POT1	RPAIN	TP53BP1	
ATM	CHEK1	FUS	MUS81	PPM1D	RPL13A	TP73	
ATR	CHEK2	GADD45A	MUTYH	PRIM1	RPL30	TREX1	
ATRIP	CHFR	GADD45B	NAE1	PRIM2	RPRM	TREX2	
ATRX	CIB1	GADD45G	NBN	PRKCG	RPS27A	TRIAP1	
ATXN3	CIDEA	GTF2E2	NCOA6	PRKDC	RRM1	TRRAP	
BAI1	CRY1	GTF2H1	NEIL1	PSMA1	RRM2	TTK	
BARD1	CRY2	GTF2H2	NEIL2	PSME4	RRM2B	TXN	
BCL2	CSNK1D	GFT2H2B	NEK11	PTEN	RUVBL1	UBA52	
BIRC5	CSNK1E	GTF2H3	NHEJ1	PTTG1	RUVBL2	UBB	
BLM	CUL4A	GTF2H4	NME2	RAD1	SEMA4A	UBE2A	
BRCA1	CUL4B	GTF2H5	NTHL1	RAD17	SESN1	UBE2B	
BRCA2	cycs	GTSE1	NUDT1	RAD18	SETX	UBE2I	
BRIP1	DCLRE1A	H2AFX	OGG1	RAD21	SF3B3	UBE2N	
BRSK1	DCLRE1B	HDAC4	OXR1	RAD23A	SHFM1	UBE2T	
BTG2	DCLRE1C	HMGB2	PALB2	RAD23B	SHISA5	UBE2V1	
BUB1	DDB1	HPRT1	PARG	RAD50	SIAH1	UBE2V2	
BUB1B	DDB2	HUS1	PARP1	RAD51	SIRT1	UNG	
CCNA2	DDIT3	IGHMBP2	PARP2	RAD51B	SLK	UPF1	
CCNB1	DDX11	ING1	PARP3	RAD51C	SLX4	USP1	
CCNB2	DKC1	INPPL1	PARP4	RAD51D	SMC1A	UVRAG	
CCNB3	DLGAP5	IP6K3	PCNA	RAD52	SMC2	VCP	
CCND1	DMC1	KAT2A	PERP	RAD54B	SMC3	WDR33	
CCND2	DNA2	KAT5	PML	RAD54L	SMC6	WRAP53	
CCND3	DNTT	KNTC1	PMS1	RAD9A	SOD1	WRN	
CCNE1	DUT	LIG1	PMS2	RB1	SPO11	WRNIP1	
CCNE2	E2F1	LIG3	PMS2l2	RBBP4	SSBP1	XAB2	
CCNG1	EI24	LIG4	PMS2p1	RBBP8	STEAP3	XPA	
CCNG2	EME1	LRIG1	PMS2p3	RBM14	SUMO1	XPC	
CCNH	EP300		PMS2p4	RECQL	SUPT3H	XRCC1	
CCNO	ERCC1	MAD2L1	PMS2p5	RECQl4	TADA3	XRCC2	

CDC25A	ERCC2	MAD2L2	PNKP	RECQL5	TAF2	XRCC3	
CDC25B	ERCC3	MBD4	POLA1	RELA	TAF5L	XRCC4	
CDC25C	ERCC4	MCM8	POLB	REV1	TCEA1	XRCC5	
CDC6	ERCC5	MDC1	POLD1	REV3L	TDG	XRCC6	
CDK1	ERCC6	MDM2	POLD3	REXO2	TDP1	XRCC6BP1	
CDK2	ERCC8	MDM4	POLDIP2	RFC1	TERF1	XRN2	
CDK4	EXO1	MGMT	POLE	RFC2	TERF2	YBX1	
CDK6	FANCA	MLH1	POLE2	RFC3	TERT	ZAK	

Gray indicates genes in the library that were not found in Traver's table

Red indicates essential genes: Hart, T., et al., High-Resolution CRISPR Screens Reveal Fitness Genes and Genotype-Specific Cancer Liabilities. Cell, 2015. 163(6): p. 1515-26

Supplementary Table 2. Screen Controls

Depletion Values of Expected Positive Controls			
Treatment	Gene	Log2FC	p Value
Chk1i	chek1	-4.61	0.0000004
	rrm1	-4.40	0.0001279
	rrm2	-3.11	0.0000027
	rpa2	-2.56	0.0042332
	luc	-0.18	0.3321634
Parpi	rbbp8	-3.34	0.0001735
	polq	-2.39	0.0028831
	brca1	-3.88	0.0000608
	brca2	-3.28	0.0205709
	rad51	-3.14	0.0018772
	luc	-0.80	0.3876199
Cisplatin	fanci	-5.68	0.0002380
	fancd2	-4.50	0.0009361
	brca1	-4.21	0.0018475
	brca2	-2.77	0.0129125
	luc	-0.90	0.0666020

Supplementary Table 3. Single agent heat map

	Gene	ShRNA Fold Depletion in each treatment group					
		Prexasertib		Talazoparib		Cisplatin	
		Log2 FC	p Value	Log2 FC	p Value	Log2 FC	p Value
Specific for Chk1i	chek1	-4.61	4.28E-07	-0.54	6.61E-01	-1.38	3.47E-01
	neil1	-2.64	5.26E-03	-1.76	1.54E-01	-1.17	1.13E-01
	dut	-3.18	8.65E-03	-1.02	1.03E-01	-2.26	3.78E-02
	triap1	-2.77	5.70E-03	-0.85	3.04E-01	-1.41	5.59E-02
	prim2	-2.89	1.11E-10	-1.42	8.37E-02	-1.38	4.15E-02
	mms19	-2.52	6.06E-03	-0.49	6.58E-02	-0.16	7.32E-02
	ube2i	-3.40	8.95E-04	-0.74	6.40E-01	-1.76	1.88E-01
	rfc1	-2.98	5.66E-03	-2.07	1.85E-02	-1.02	4.61E-01
	tipin	-2.28	2.86E-03	-0.98	5.61E-01	-1.17	2.22E-01
	pole	-2.23	9.38E-04	-1.37	1.83E-01	-1.85	1.36E-01
	pold1	-2.73	2.25E-03	-1.38	1.67E-03	-1.05	3.76E-02
Specific for PARPi	pnkp	-0.95	3.80E-01	-2.66	7.30E-08	-1.23	3.14E-01
	rbbp8	-0.54	3.21E-01	-3.34	1.73E-04	-2.28	1.15E-02
	ube2n	-1.30	2.89E-01	-3.88	5.88E-03	-2.39	3.35E-02
	nae1	-0.41	4.04E-01	-2.59	2.29E-02	-2.29	4.26E-03
	rad23b	-1.29	1.63E-01	-3.06	3.53E-03	-1.16	1.22E-01
	mdc1	-1.14	6.32E-02	-3.72	2.50E-05	-2.08	5.29E-02
	nbn	-0.48	4.50E-01	-3.31	8.55E-04	-1.50	4.34E-03
	rnf8	-1.47	1.78E-01	-2.62	3.72E-03	-1.11	3.65E-02
	atm	-0.80	5.18E-02	-3.13	2.36E-03	-1.50	6.92E-02
Specific for cisplatin	dmc1	-1.36	1.78E-02	-0.78	1.95E-01	-4.34	6.06E-03
	rad9a	-1.61	2.45E-01	-1.25	3.05E-02	-4.70	2.74E-05
	nthl1	-1.44	2.44E-01	-1.31	1.11E-01	-2.64	4.88E-03
	fanci	-1.36	1.35E-02	-1.33	1.69E-01	-5.68	2.38E-04
	brip1	-0.74	1.28E-01	-0.25	6.44E-01	-2.50	8.91E-05
	cdk1	-1.62	8.43E-02	-0.77	1.70E-01	-3.26	7.54E-03
	topbp1	-1.26	2.74E-01	-2.09	7.25E-05	-3.23	1.95E-05
	vcp	-1.56	4.01E-02	-0.75	5.43E-01	-2.63	1.23E-03
	mad2l1	-2.05	2.93E-02	-1.93	7.34E-02	-3.63	9.00E-03
	terf1	-2.02	7.00E-02	-2.22	1.90E-02	-2.51	9.15E-03
Common to all three	rfc4	-2.88	3.40E-04	-3.32	3.09E-04	-2.94	2.50E-03
	rfc2	-3.94	4.59E-04	-4.09	1.15E-02	-7.24	6.72E-04
	pcna	-2.80	1.70E-02	-4.48	2.89E-03	-3.23	1.50E-02
	polg	-2.96	9.93E-03	-4.35	4.07E-03	-5.16	5.77E-03
	rfc5	-4.22	3.17E-04	-3.20	1.22E-02	-3.71	7.27E-03
	wdr33	-2.97	1.14E-03	-2.67	2.02E-02	-2.71	6.58E-03
	rrm2	-3.11	2.74E-06	-2.52	3.84E-02	-2.46	4.82E-02
	fancd2	-2.28	4.34E-02	-2.42	5.75E-02	-4.50	9.36E-04

Log2 FC

-0.16 (Maximum)

=2.24 (Median)

=7.24 (Minimum)

p Value

6.61E-01 (Maximum)

1.96E-02 (Median)

1.11E-10 (Minimum)

Supplementary Table 4. Screen results

gene_id	A2_Cis_Log2FC	A2_Cis_LogP	A2_Cis_P	A2_LY_Log2FC	A2_LY_LogP	A2_LY_P	A2_BMN_Log2F	A2_BMN_Log	A2_BMN_P
abcf2	-0.660713527	-0.3149676	0.48420853	-0.750317971	-1.5857134	0.02595892	-0.559523512	-0.40258845	0.395741
abl1	-1.606534899	-1.6141099	0.02431588	-2.008543768	-0.922444	0.11955177	-1.748755891	-1.00998241	0.097728
aifm1	-1.425924479	-1.2029261	0.06267206	-1.723829941	-1.5550103	0.02786055	-0.631623794	-2.32407976	0.004742
alkbh1	-2.210894303	-1.5660303	0.0271625	-0.682752106	-0.5181059	0.30331517	-0.741256527	-1.08079351	0.083025
ankrd17	-1.300845965	-1.4963445	0.03189007	-1.430797011	-1.4484811	0.03560565	-3.093265217	-1.44809895	0.035637
antxr1	-0.714853605	-0.1950747	0.63815375	-1.536504656	-0.7564617	0.17520168	-3.014728855	-1.84236673	0.014376
apaf1	-0.992074947	-1.1878759	0.06488198	-0.829799875	-0.514488	0.30585247	-1.283120311	-1.09162522	0.080979
apex1	-1.27672876	-0.663929	0.21680586	-2.574236839	-1.8208223	0.01510698	-3.308661024	-2.06308544	0.008648
apex2	-1.269937673	-0.6395679	0.22931481	-0.695570718	-1.1814263	0.06585272	-1.309434402	-0.64805063	0.224879
aptx	-1.021330561	-0.7273578	0.18734506	-0.492198077	-1.030648	0.09318628	-0.941649984	-1.95122516	0.011189
asf1a	-1.092163501	-0.3018325	0.49907699	-1.504283229	-3.1081467	0.00077957	-1.653380518	-1.08989817	0.081302
atm	-1.50405223	-1.1599271	0.06919471	-0.800019851	-1.2856536	0.05180199	-3.129535105	-2.62675819	0.002362
atr	-3.328995103	-3.3025573	0.00049824	-1.478594052	-1.5126102	0.03071778	-4.848167959	-5.51402146	3.06E-06
atrip	-1.501231744	-0.5419099	0.28713764	-0.890078582	-0.385894	0.41125004	-2.341446951	-1.06817982	0.085471
atrx	-2.035335891	-1.0646152	0.08617569	-1.020408562	-0.5144975	0.30584581	-0.880085889	-0.32642113	0.471606
atxn3	-1.044329555	-0.8977184	0.12655566	-1.520487253	-0.8087638	0.15532316	-0.264787855	-0.29510128	0.506872
bai1	-0.794384636	-0.322537	0.4758423	-0.521857658	-0.2951013	0.50687248	-1.132577602	-1.55133031	0.028098
bard1	-1.716405904	-1.6955651	0.02015742	-0.749420331	-0.5871976	0.25870353	-2.33817712	-3.15486644	0.0007
bcl2	-0.963144969	-0.4988972	0.31703176	-0.373936464	-0.5076809	0.31068418	-2.921264546	-1.18367689	0.065512
birc5	-0.666917092	-1.4502358	0.03546208	-0.906674351	-1.286624	0.05168637	-0.339292825	-0.76187847	0.17303
blm	-1.160868919	-1.21976	0.06028927	-0.486654447	-0.8991172	0.12614871	-1.145958956	-0.65535216	0.22113
brca1	-4.210629686	-2.7334061	0.00184754	-1.366408042	-1.6006206	0.025083	-3.875637663	-4.21631504	6.08E-05
brca2	-2.769069607	-1.8889882	0.01291254	-1.789091741	-1.1797768	0.0661033	-3.280226444	-1.68674686	0.020571
brip1	-2.503855883	-4.0499116	8.9143E-05	-0.742842076	-0.8926405	0.12804407	-0.245108272	-0.19142384	0.643541
brsk1	-1.196913533	-0.4082124	0.39064981	-0.57988903	-0.1814739	0.65845502	-0.944232998	-0.8143393	0.153342
btg2	-0.773805085	-0.584321	0.26042278	-1.262359822	-0.5605526	0.27507267	-0.806006499	-0.96588044	0.108173
bub1	-2.529306809	-1.5773168	0.02646569	-1.338807577	-2.0028486	0.00993462	-2.291300251	-1.97117436	0.010686
bub1b	-2.262516088	-1.2281379	0.05913738	-0.950289238	-0.2809453	0.52366639	-1.869321477	-0.79993182	0.158514
ccna2	-2.31588921	-1.1496422	0.07085293	-0.525564783	-0.1300081	0.74129647	-0.652677693	-0.31802287	0.480814
ccnb1	-1.801646971	-0.8255037	0.14945015	-1.62851266	-1.751812	0.01770875	-1.121905455	-0.6890814	0.204606
ccnb2	-0.122637578	-0.2924117	0.51002124	-1.059944512	-0.7663743	0.17124808	-0.217365901	-0.06867322	0.853742
ccnb3	-1.567379485	-0.5943583	0.25447302	-1.416443445	-1.0177794	0.09598882	-1.238752482	-0.71610024	0.192265
ccnd1	-1.298970322	-0.6238593	0.23776103	-1.702720683	-1.158391	0.06943988	-0.809487449	-0.54282302	0.286535
ccnd2	0.012041108	-0.2656075	0.542491	-0.267312475	-0.2065327	0.62153748	-0.645025368	-0.31127365	0.488345
ccnd3	-0.514150662	-0.7142106	0.19310318	-0.342881013	-0.246688	0.56664625	-0.946999013	-0.39612678	0.401674
ccne1	-1.319007341	-0.9430691	0.11400683	-0.476827513	-0.0673267	0.85639345	-0.901778445	-0.69177233	0.203342
ccne2	-2.997251831	-1.4319081	0.03699065	-0.734135247	-1.1593971	0.06927921	-0.454255922	-0.46799143	0.340415
ccng1	-1.266206959	-0.5505902	0.28145556	-1.652864598	-1.4544154	0.03512243	-0.977474181	-0.77912812	0.166292
ccng2	-1.270399342	-1.1178316	0.07623746	-1.479367225	-0.6631272	0.2172065	-0.901079435	-1.66453864	0.02165
ccnh	-2.078316211	-1.0236786	0.09469376	-2.340992441	-1.2526428	0.05589297	-0.639336886	-0.87623629	0.132973
ccno	-2.185134524	-1.1868737	0.06503188	-0.856014068	-0.8106128	0.15466328	-1.53251807	-0.57713786	0.264766
cdc25a	-0.832044086	-0.7973236	0.15946904	-0.75032751	-0.1984516	0.63321097	-0.945068596	-0.49493044	0.319941
cdc25b	-0.562044076	-0.5242878	0.29902827	0.11531965	-0.0084739	0.98067735	-1.245207672	-0.91618731	0.121287
cdc25c	-0.815581136	-0.7044455	0.19749428	-1.613112676	-0.8842565	0.13053995	-0.933849564	-0.4261985	0.374802
cdc6	-1.039535297	-0.2575983	0.55258827	-1.550740881	-0.7927961	0.16114018	-1.06295946	-0.36456805	0.431948
cdk1	-3.258870802	-2.1225574	0.00754124	-1.623690575	-1.074156	0.08430319	-0.774514808	-0.77020058	0.169746
cdk2	-2.324643247	-0.9578223	0.11019901	0.533171164	-0.0003181	0.99926782	-2.55707277	-1.44863667	0.035593
cdk4	-0.627085222	-0.1039301	0.78717247	-2.073770807	-1.1381955	0.07274523	-0.823023886	-0.47980641	0.331279
cdk6	-1.125942092	-0.3182241	0.48059128	-0.274375116	-0.1353092	0.7323029	0.45316699	-0.22835726	0.591075
cdk7	-1.538035167	-0.5448498	0.28520042	-1.527832624	-1.2281379	0.05913738	-2.634014148	-1.45570009	0.035019
cdkn1a	-1.75168708	-0.8882612	0.12934178	-0.674976028	-0.7949563	0.16034067	-1.032267568	-0.9282895	0.117953
cdkn2a	-0.914620864	-1.0776149	0.08363442	-1.042000255	-1.0658429	0.08593244	-1.385222879	-0.89727384	0.126685
cdkn2b	-0.428885404	-0.4721929	0.33713751	-1.343520122	-0.9516975	0.11176415	-0.340012232	-1.05782152	0.087534
cdkn2d	-2.856850941	-1.7517859	0.01770982	-0.302992286	-0.3465217	0.45027544	-1.629095137	-0.95932224	0.109819
cdt1	-0.790893462	-0.3274548	0.47048439	-0.494269818	-0.5542312	0.27910576	-0.647770684	-0.84830909	0.141805
cebpg	-1.683292937	-0.7809155	0.1656092	-1.322400568	-0.514119	0.30611246	-1.817964184	-0.92536324	0.118751
cenpf	0.180959917	-0.0137745	0.96878072	-1.558293707	-0.5414328	0.2874532	-1.097042003	-0.34419966	0.452689
cetn2	-0.797343761	-1.6038582	0.0248967	-2.079266651	-3.290541	0.00051222	-0.439872852	-0.90655531	0.124007
chaf1a	-0.532520137	-0.1219011	0.75526416	-1.980697727	-1.3548152	0.04417584	-1.438592406	-0.58175171	0.261968

chaf1b	-2.454058572	-1.2063025	0.0621867	-1.114724714	-0.6088331	0.24613133	-0.867731962	-0.35742512	0.439112
chek1	-1.376684248	-0.4594677	0.34716207	-4.608578278	-6.3681697	4.2838E-07	-0.538714232	-0.17961878	0.661274
chek2	-0.702994988	-0.8695479	0.13503679	-1.901968201	-0.9828389	0.10403061	-0.945216823	-0.46029733	0.3465
chfr	-3.600987141	-1.7637503	0.01722859	-1.088271147	-0.4188902	0.38116221	-2.656402293	-1.18001793	0.066067
cib1	-1.455572648	-0.5063328	0.31165002	-1.379671545	-0.6855309	0.20628569	-2.293278567	-1.05138812	0.088841
cidea	-0.73717145	-0.9282895	0.11795341	-1.377246689	-1.0273614	0.09389417	-0.257575327	-0.89418443	0.12759
cry1	-1.916446037	-0.8148873	0.15314848	-1.006131006	-1.0578215	0.08753434	-1.403181759	-0.63035069	0.234234
cry2	-1.492520564	-1.3894094	0.04079347	-1.462749508	-2.1184754	0.00761245	-1.821164534	-0.98414812	0.103717
csnk1d	-0.865599251	-0.3112736	0.48834456	-1.1243807	-0.9096535	0.12312506	-1.079933922	-1.32455525	0.047364
csnk1e	-1.303302222	-0.757567	0.17475635	-0.862386866	-1.0658462	0.08593178	-1.140114005	-0.781394	0.165427
cul4a	-1.914835235	-1.9606649	0.01094801	-1.681806363	-2.1353516	0.00732231	-1.968054717	-1.1482184	0.071086
cul4b	-2.09293491	-1.1596167	0.06924419	-1.162890437	-1.2360293	0.05807252	-0.957501647	-0.67601679	0.210855
cycs	-0.565934921	-0.4332796	0.36874012	-0.420880944	-1.2284083	0.05910057	-0.22716677	-0.13093711	0.739712
dclre1a	-0.951750579	-2.135089	0.00732674	-0.952072151	-1.258457	0.05514968	-1.338286136	-1.27441918	0.053159
dclre1b	-1.063372516	-0.4779231	0.33271844	-1.118765651	-0.433639	0.36843513	-0.615359008	-0.37109294	0.425507
dclre1c	-3.169586275	-1.6108925	0.02449669	-2.31802797	-1.4658039	0.03421339	-1.690797976	-0.69010252	0.204126
ddb1	-2.462818433	-1.6406662	0.02287356	-2.381677957	-1.4319081	0.03699065	-2.311705422	-0.98336549	0.103905
ddb2	-1.332406338	-0.7071756	0.19625668	-1.309352049	-1.9816617	0.0104313	-1.039271053	-0.70317084	0.198075
ddit3	-2.079189909	-1.1971698	0.06350826	-1.496164005	-0.8861977	0.12995779	-1.373342723	-1.3494157	0.044728
ddx11	-0.475019761	-0.3153262	0.48380888	-1.002352963	-0.3245456	0.47364655	-0.968940159	-2.13303458	0.007361
dkc1	-1.017481552	-1.3120511	0.04874711	-1.831860123	-0.7548988	0.17583334	-1.141021566	-1.95595199	0.011067
dlgap5	-1.376305273	-1.1449729	0.07161881	-0.358027066	-0.1084988	0.77893504	-0.984913401	-0.35944047	0.437079
dmc1	-4.341150194	-2.2174028	0.00606174	-1.356832821	-1.7499312	0.01778561	-0.779686407	-0.70890322	0.195478
dna2	-1.901143753	-0.6434634	0.2272671	-1.054797075	-0.4840902	0.32802719	-2.009538586	-1.59542541	0.025385
dntt	-1.942735846	-0.9198086	0.12027944	-1.562953816	-0.7161002	0.19226479	-1.078664292	-0.45239401	0.352863
dut	-2.255151752	-1.4228467	0.03777054	-3.175948865	-2.0630854	0.00864798	-1.021021858	-0.9854866	0.103398
e2f1	-1.04882645	-0.4933424	0.32111278	-2.205338148	-1.9387118	0.01151564	-0.770653749	-0.66140139	0.218071
ei24	-0.672066024	-0.6102793	0.24531308	-0.679677921	-0.3848039	0.41228362	-0.974824404	-1.17647985	0.066607
eme1	-2.570407244	-1.2384235	0.05775326	-1.397924993	-0.9279661	0.11804127	-1.391845872	-0.99127108	0.10203
ep300	-0.37097683	-0.4770541	0.3333849	-0.950212144	-0.2543542	0.55673151	-0.705342292	-0.29623731	0.505548
ercc1	-1.584690238	-0.6251323	0.23706514	-1.361606189	-1.0894153	0.08139256	-2.18341785	-1.65834284	0.021961
ercc2	-1.165745147	-0.863658	0.13688064	-1.276139122	-0.7663876	0.17124283	-1.006873072	-0.56622821	0.271501
ercc3	-1.082999227	-0.8131055	0.15377809	-0.43245748	-0.639305	0.22945368	-0.412383668	-0.23681656	0.579673
ercc4	-1.051810612	-0.6592124	0.21917326	-0.372308936	-0.5155312	0.30511867	-0.523569847	-0.81159019	0.154316
ercc5	-0.468119633	-1.4432212	0.03603951	-1.863128456	-1.3176565	0.04812198	-0.481255393	-1.12406571	0.075151
ercc6	-1.038583281	-0.7081319	0.19582501	-1.296535601	-0.4600948	0.34666114	-0.807936217	-0.58190906	0.261873
ercc8	-0.275662221	-0.1942876	0.63931128	-1.157982525	-0.8631695	0.13703468	-0.524253108	-0.15919735	0.693111
exo1	-0.017534847	-0.7573873	0.17482868	-0.689016275	-0.9941524	0.10135556	-1.049418777	-0.33412227	0.463316
fanca	-1.247363003	-0.5115585	0.30792257	-0.60699913	-0.1617245	0.68908934	-1.058565755	-0.61417597	0.243122
fancb	-1.45223853	-0.597295	0.25275804	-1.822060275	-1.1697406	0.06764869	-2.66106728	-1.30451969	0.0496
fancc	-0.633856363	-0.6796397	0.209103	-1.17913623	-1.434552	0.03676614	-0.868231831	-1.06906021	0.085298
fancd2	-4.500404317	-3.028697	0.00093606	-2.282391377	-1.3625831	0.04339272	-2.420795575	-1.24002048	0.057541
fance	-1.117704416	-0.8843663	0.13050696	-1.393039331	-0.6967862	0.2010082	-1.170163927	-0.50998205	0.309042
fancf	-1.185717121	-0.8322815	0.14713585	-0.049631266	-0.195758	0.63715045	-0.866250852	-0.4536817	0.351818
fancg	-1.210635356	-0.8683803	0.13540032	-1.433849027	-1.5282055	0.02963429	-2.392091135	-1.22813794	0.059137
fanci	-5.680439136	-3.6234032	0.00023801	-1.360168734	-1.8700468	0.01348818	-1.325828642	-0.77274694	0.168754
fancl	-0.585958093	-0.2883805	0.51477739	-0.044717647	-0.2138102	0.61120905	-0.479107032	-0.51247665	0.307272
fancm	-1.083997583	-0.6890639	0.20461434	-1.331583312	-2.5171805	0.00303962	-2.408863211	-1.32335283	0.047495
fen1	-1.438678915	-1.4625388	0.03447158	-0.342586293	-0.7874165	0.16314867	-1.040698441	-0.66852592	0.214523
foxn3	-0.715789695	-0.1821494	0.65743162	-1.450609142	-0.8007842	0.15820341	-0.930965889	-0.21060081	0.615743
fus	-1.241493075	-0.795774	0.16003905	-0.729393482	-0.4696954	0.33908187	-0.169139581	-0.207214	0.620563
gadd45a	-0.728304481	-0.9086526	0.12340915	-0.776116137	-0.3072249	0.49291847	-0.631708763	-0.37468332	0.422004
gadd45b	-0.879230755	-0.6197104	0.24004332	-1.181152914	-0.4341027	0.36804195	-1.028562234	-0.31278397	0.486649
gadd45g	-2.182041189	-0.8596035	0.13816452	-1.485624113	-0.564238	0.27274827	-1.047525569	-0.54630979	0.284243
gtf2e2	-1.829874208	-1.0361541	0.09201231	-1.789357095	-1.1161686	0.07652995	-1.561450331	-1.61249567	0.024406
gtf2h1	-2.239524962	-1.040042	0.09119227	-1.591478137	-1.0160219	0.09637805	-1.71745363	-1.02542487	0.094314
gtf2h2	-0.8815885	-0.4676008	0.34072121	-0.760238068	-0.2478698	0.56510641	-0.436830505	-0.31262407	0.486828
gtf2h2b	-1.618874683	-2.0941849	0.00805036	-0.838028016	-1.0983625	0.07973288	-1.497964728	-0.63784906	0.230224
gtf2h3	-2.412094642	-1.473112	0.03364248	-1.159497087	-0.5996982	0.25136324	-1.085607915	-0.2910224	0.511655
gtf2h4	-0.38279593	-0.4294846	0.37197638	-0.402945602	-0.3999362	0.39816569	-0.499458436	-0.4468478	0.357398
gtf2h5	-1.211245186	-1.384039	0.04130104	-0.94278995	-1.192677	0.06416867	-1.091906163	-0.75557816	0.175558
gtse1	-0.840405931	-0.509678	0.30925874	-1.569190726	-0.8005982	0.15827117	-0.700021634	-0.60172719	0.250192

h2afx	-1.881459259	-0.984255	0.10369194	-0.566215886	-0.3601552	0.43635982	-1.539898338	-1.65875189	0.021941
hdac4	-1.164476364	-0.3168955	0.48206379	-0.166002945	-0.1613189	0.68973316	-0.348455975	-0.4386451	0.364213
hmgb2	-2.243392884	-2.1867668	0.00650479	-0.458727374	-0.2004356	0.63032487	-1.69178167	-1.00502704	0.098849
hprt1	-1.983605891	-0.7002744	0.19940022	-1.496933395	-1.858463	0.01385278	-1.32499278	-0.50826463	0.310267
hus1	-1.57490181	-2.0391316	0.00913836	-1.047471328	-1.2798685	0.05249664	-1.677882161	-0.72885558	0.1867
ighmbp2	-1.856172002	-1.1897545	0.06460194	-0.42603665	-0.6876255	0.20529316	-0.629434613	-0.32750646	0.470428
ing1	-0.249622293	-0.4540157	0.35154771	-1.132477748	-0.6670602	0.21524834	-1.785940656	-1.71082833	0.019461
inppl1	-1.133764102	-1.0754954	0.08404359	-2.007592065	-0.817137	0.15235722	-1.612873325	-1.92269493	0.011948
ip6k3	-0.3354411	-0.4568073	0.34929529	-1.149282411	-0.4623318	0.34488014	-2.050652384	-0.7745234	0.168065
kat2a	-1.538573492	-0.4859021	0.32666146	-1.516209707	-0.7682406	0.17051376	-0.767761802	-0.24565092	0.568001
kat5	-0.52855112	-0.34244	0.4545273	-0.902008573	-1.2261993	0.05940194	-1.050099443	-0.40640112	0.392282
kntc1	-1.093171935	-0.3695342	0.42703734	-0.190946068	-0.3697169	0.42685764	-0.052540341	-0.14037965	0.723803
lig1	-0.824895626	-0.9387183	0.11515471	-0.55839182	-0.5630545	0.27349256	-2.45059677	-1.14193996	0.072121
lig3	-2.76899553	-1.1071344	0.07813859	-1.018763034	-0.6051873	0.24820625	-2.781882664	-1.58391106	0.026067
lig4	-0.826634497	-0.3195814	0.47909161	-1.008441361	-0.5799932	0.26303091	-0.851602306	-0.39263639	0.404915
lrig1	-2.284899096	-0.9292334	0.11769734	-1.239536396	-0.9220686	0.11965514	-0.415742001	-0.51272775	0.307095
luc	-0.904163165	-1.1765127	0.06660201	-0.180165381	-0.4786483	0.33216335	-0.799719889	-0.41159392	0.38762
mad2l1	-3.634730577	-2.0457031	0.00900113	-2.054063484	-1.5334431	0.02927905	-1.925586388	-1.1340618	0.073441
mad2l2	-0.462839168	-0.4990293	0.31693538	-0.468582832	-0.3021451	0.49871785	-0.624124336	-0.27113869	0.535626
mbd4	-1.210859069	-0.4873009	0.32561101	-1.364668311	-0.9279854	0.11803602	-0.705750332	-0.44425941	0.359535
mcm8	-1.099986833	-0.7120025	0.19408749	-1.203857027	-2.9028295	0.00125075	-1.11461408	-0.43232559	0.369551
mdc1	-2.082494428	-1.2768952	0.05285728	-1.140372243	-1.1996115	0.06315221	-3.722678197	-4.60236474	2.5E-05
mdm2	-1.287256787	-0.6779245	0.20993048	-1.934882552	-1.8461539	0.01425103	-1.673081184	-1.12300953	0.075334
mdm4	-1.185907983	-0.369869	0.42670818	-1.193743206	-0.5902495	0.25689195	-0.582545966	-0.7663292	0.171266
mgmt	-0.250932845	-0.8230716	0.15028941	-0.936910596	-0.4252059	0.37565925	-0.748138784	-0.85741673	0.138862
mlh1	-0.434371437	-0.8988194	0.12623524	-0.705066378	-0.6472984	0.22526911	-0.522486506	-0.80250927	0.157576
mlh3	-0.868169622	-0.9367046	0.11568988	-1.871200953	-1.0403352	0.09113071	-0.38708813	-0.57258269	0.267558
mms19	-0.160921384	-1.1354774	0.07320195	-2.517701892	-2.2174028	0.00606174	-0.485767577	-1.18169973	0.065811
mnat1	-1.568389523	-1.5712067	0.02684066	-0.871343977	-0.5354174	0.29146243	-1.203842256	-1.19872228	0.063282
mpg	-0.967516691	-0.5952116	0.25397348	-1.011849088	-0.2732787	0.53299272	0.056795758	-0.66120223	0.218171
mre11a	-2.082254319	-1.7485068	0.01784404	-2.490461442	-1.7869753	0.01633145	-3.161296689	-2.25943243	0.005503
msh2	-0.118241847	-0.4526206	0.35267882	-0.082302669	-0.4082124	0.39064981	-0.197433951	-0.40683192	0.391894
msh3	-3.021043203	-1.4661063	0.03418957	-1.162697366	-1.3427997	0.04541511	-1.143507052	-2.0035356	0.009919
msh4	-0.822078528	-0.3656207	0.43090283	-0.768929056	-0.5328654	0.29318018	-1.367721392	-0.3972868	0.400602
msh5	-3.301479813	-1.4701437	0.03387321	-1.606132745	-0.7186035	0.19115976	-2.389099465	-0.94879857	0.112513
msh6	-0.201499149	-0.3062119	0.49406955	-1.03267427	-0.278102	0.52710606	-1.421918063	-0.55987977	0.275499
mtor	-0.173137276	-0.0209109	0.95299155	-0.596886134	-0.3831852	0.41382315	-0.451254384	-0.15451718	0.70062
mus81	-0.038241299	-0.0703002	0.85054985	-0.196076441	-0.1574075	0.69597317	-0.30054416	-0.48482187	0.327475
mutyh	-1.888825353	-0.7421693	0.1810634	-0.148070628	-0.9563943	0.11056196	-1.354224461	-1.54963047	0.028208
nae1	-2.287866565	-2.3707759	0.00425818	-0.406102357	-0.3935516	0.40406238	-2.589586332	-1.63994079	0.022912
nbn	-1.497186593	-2.3628853	0.00433625	-0.477901459	-0.3465456	0.45025069	-3.305294464	-3.06794725	0.000855
ncoa6	-3.218821177	-1.697212	0.02008112	-1.394307488	-1.6458667	0.0226013	-1.621562227	-1.42956088	0.037191
neil1	-1.169645728	-0.9470058	0.11297809	-2.637498943	-2.2787782	0.00526286	-1.760246381	-0.81134416	0.154403
neil2	-0.973926062	-0.6349387	0.23177216	-2.302312268	-1.5330197	0.0293076	-1.395786783	-0.66588932	0.215829
nek11	-0.981259286	-0.4712242	0.33789034	-0.726523532	-0.6635935	0.2169734	-0.716300288	-0.79043084	0.16202
nhej1	-1.055394605	-0.9629306	0.10891041	-1.523807536	-1.6843213	0.0206861	-2.150059428	-1.01325922	0.096993
nme2	-1.048205948	-0.4790923	0.33182391	-1.207591325	-0.4849719	0.32736188	-1.002820807	-0.60305309	0.249429
nthl1	-2.63635012	-2.3112213	0.00488403	-1.442080399	-0.6130864	0.24373257	-1.310990231	-0.95280741	0.111479
nudt1	-0.709271333	-0.4554113	0.35041987	-0.995899641	-0.2504735	0.56172856	-0.567944565	-0.1307678	0.740001
ogg1	-1.254035498	-0.4479903	0.35645909	-0.226816175	-0.2084232	0.61883779	-1.568262535	-0.77777461	0.166811
oxr1	-0.639988448	-0.5135236	0.30653239	-0.336986277	-0.2416243	0.57329182	-0.879210037	-0.82432333	0.149857
palb2	-2.833960519	-1.7845816	0.01642171	-1.566368157	-0.7278688	0.18712474	-3.159476071	-1.61793329	0.024103
parg	-0.842620144	-0.1630048	0.6870609	-0.924957164	-0.4049854	0.39356332	-1.016432822	-0.43627918	0.366202
parp1	-0.433247108	-0.3590695	0.43745213	-0.764424938	-0.5897536	0.25718543	0.828381445	-0.00186308	0.995719
parp2	-0.813637971	-0.8801783	0.13177156	-0.666575947	-0.2384517	0.57749514	-1.311407306	-0.69859386	0.200173
parp3	-1.756568096	-0.8815002	0.13137108	-0.902484255	-1.0258703	0.09421709	-1.013736183	-0.38680695	0.410386
parp4	-1.784935388	-0.7828988	0.16485463	-1.964168894	-1.5952526	0.02539495	-1.957614907	-1.19561835	0.063736
pcna	-3.234560919	-1.8227956	0.0150385	-2.799975087	-1.7703396	0.01696916	-4.476603898	-2.5390761	0.00289
perp	-1.619193163	-0.6117978	0.24445682	-2.184234914	-1.0530435	0.0885027	-0.839084578	-0.31725767	0.481662
pml	-1.12239525	-0.8957289	0.12713676	-0.34045334	-0.8419978	0.14388059	-1.126604071	-1.22369704	0.059745
pms1	-0.321900227	-1.0020602	0.09952674	-1.13922324	-0.8762363	0.13297308	-0.735022092	-0.92984612	0.117531
pms2	-0.953560596	-0.6016093	0.25025958	-0.801669901	-0.7104891	0.19476499	-1.755740364	-0.83645442	0.145729

pms2l2	-0.646333808	-1.6762962	0.02107191	-0.758623589	-0.7648752	0.17184023	-1.404557094	-0.98795288	0.102813
pms2p1	-0.315161159	-0.3683559	0.42819744	-0.363092464	-0.2491785	0.563406	-2.015143066	-1.26978388	0.05373
pms2p3	-1.242850227	-0.3609972	0.43551467	-1.195017301	-0.4368592	0.36571334	-1.517711782	-0.92016147	0.120182
pms2p4	-0.613037928	-0.5171647	0.30397321	-1.118357527	-0.3913651	0.40610174	-1.21542677	-0.51660684	0.304364
pms2p5	-0.718173016	-0.4551078	0.35066478	-1.148717095	-0.5641163	0.27282468	-1.787768981	-1.61295906	0.02438
pnkp	-1.227633871	-0.5026083	0.31433428	-0.949833519	-0.4203445	0.37988797	-2.658829511	-7.13654296	7.3E-08
pola1	-3.319909358	-1.9560573	0.01106478	-3.4403821	-3.9034188	0.00012491	-1.288015398	-0.57255577	0.267574
polb	-1.368515213	-1.0029382	0.09932573	-0.972432163	-0.2377912	0.57837399	-1.07804121	-1.46896631	0.033965
pold1	-1.04768943	-1.4247023	0.03760951	-2.729796251	-2.6470006	0.00225424	-1.383951013	-2.77605518	0.001675
pold3	-2.379462694	-1.7884107	0.01627756	-2.401567432	-1.5893527	0.0257423	-2.153431626	-1.02933615	0.093468
poldip2	-1.120392798	-0.9783014	0.10512321	-1.395229063	-1.4682463	0.03402152	-0.837616283	-0.40243474	0.395882
pole	-1.847912425	-0.8676452	0.13562971	-2.230604876	-3.0279512	0.00093767	-1.37131556	-0.73846582	0.182614
pole2	-2.49407313	-1.5196403	0.03022454	-3.599010749	-3.4475696	0.0003568	-1.73840809	-1.02377116	0.094674
polg	-5.157600357	-2.2386115	0.00577283	-2.962275039	-2.0030207	0.00993069	-4.347516945	-2.39062989	0.004068
polg2	-0.510067828	-0.1722185	0.67263812	-0.903430909	-0.6979448	0.20047267	-0.623402668	-0.30022902	0.500923
polh	-0.684349753	-0.1866906	0.65059302	-0.681444693	-0.34061	0.45644667	-0.265491946	-0.04381124	0.904042
poli	-1.590242842	-0.4940974	0.32055504	-0.468904048	-0.7618785	0.17303005	-0.631851892	-0.33645203	0.460838
polk	-0.798242128	-0.6734576	0.21210083	-0.464201801	-0.3139749	0.48531654	-1.633509852	-1.0545954	0.088187
poll	-0.78373063	-0.5613766	0.27455126	-0.88503372	-0.8880122	0.12941596	-0.835823542	-0.58454296	0.26029
polm	-0.83961728	-0.7778355	0.16678789	-0.920990761	-0.9252893	0.11877108	-1.794871609	-0.97837994	0.105104
poln	-1.65282028	-0.5616177	0.27439885	-1.384200149	-0.7243413	0.18865081	-1.454099196	-0.9484642	0.112599
polq	-1.928705482	-1.3525638	0.04440544	-3.111673486	-2.5390761	0.00289017	-2.389584475	-2.54013784	0.002883
polr2g	-1.922812499	-1.1334677	0.07354147	-0.859088463	-0.5323668	0.29351698	-1.720731467	-1.16275611	0.068745
pot1	-1.113052368	-1.0548778	0.08812967	-0.547858847	-0.6834884	0.20725813	-1.370302092	-0.90500702	0.124449
ppm1d	-1.689118278	-0.9189615	0.12051427	-0.404279507	-1.1192976	0.07598054	-0.503915714	-0.53286539	0.29318
prim1	-0.665389858	-0.3601955	0.43631935	-2.513620463	-1.4870035	0.03258341	-1.543106653	-0.69462036	0.202013
prim2	-1.384492594	-1.3824294	0.0414544	-2.893710665	-9.9528951	1.1146E-10	-1.421901261	-1.07710569	0.083733
prkcg	-0.790147566	-0.7984031	0.15907317	-1.045271688	-0.7948601	0.16037618	-0.644626015	-0.86187753	0.137443
prkdc	-0.952240338	-0.7678739	0.17065778	-1.767562025	-1.0758303	0.08397881	-0.795522829	-1.38923832	0.04081
psma1	-1.870079257	-0.9388929	0.11510842	-1.283980178	-1.1866329	0.06506794	-1.995726189	-1.37615234	0.042058
psme4	-0.811175721	-1.8353877	0.01460872	-1.021912401	-0.5296887	0.29533254	-1.897255881	-1.18049167	0.065995
pten	-2.19772724	-1.3530135	0.04435949	-1.326860961	-1.497189	0.03182812	-1.510520614	-1.15564135	0.069881
pttg1	-1.372995918	-0.758631	0.17432873	-1.55494442	-0.7723692	0.16890043	-0.422844333	-0.37775103	0.419034
rad1	-2.770699375	-1.5195088	0.03023369	-1.316559176	-0.5051222	0.31252	-1.631109413	-0.88955442	0.128957
rad17	-1.727536249	-1.4593784	0.03472335	-1.169159896	-1.6330484	0.02327832	-2.547694981	-1.92587517	0.011861
rad18	-1.553401079	-1.889422	0.01289965	-0.650565752	-1.8708919	0.01346196	-1.532378822	-2.98489598	0.001035
rad21	-4.60642247	-2.2787782	0.00526286	-2.03822896	-2.7627793	0.00172672	-2.632829293	-1.46610632	0.03419
rad23a	-0.195386925	-0.2191009	0.60380836	-1.585669066	-1.692068	0.02032039	-0.973738777	-0.24794369	0.56501
rad23b	-1.163004481	-0.9151082	0.12158831	-1.28843204	-0.7887443	0.1626506	-3.059957921	-2.45179893	0.003533
rad50	-1.790871643	-1.0570535	0.08768927	-1.21268385	-0.525272	0.29835136	-1.977731826	-1.9666906	0.010797
rad51	-2.752962019	-1.6219434	0.02388123	-1.494740685	-0.7383521	0.18266186	-3.135789342	-2.72648511	0.001877
rad51b	-1.104717709	-0.5843357	0.26041399	-0.470348398	-0.2500107	0.56232744	-1.326367712	-0.69420922	0.202204
rad51c	-4.713779698	-2.1016731	0.00791274	-1.5509667	-0.7745234	0.16806474	-5.778748647	-3.8915877	0.000128
rad51d	-0.876076033	-0.6657856	0.21588098	-1.126943777	-0.3262833	0.47175525	-1.062887588	-1.29508316	0.050689
rad52	-2.757870272	-1.274206	0.0531856	-2.214593456	-1.2396606	0.05758898	-2.671581296	-1.43190807	0.036991
rad54b	-2.321372538	-2.2486746	0.0056406	-1.781120461	-0.8217758	0.15073851	-0.681650396	-0.36179203	0.434718
rad54l	-1.217419914	-0.5873965	0.2585851	-2.399762928	-1.2933961	0.05088666	-1.257830603	-0.70089771	0.199114
rad9a	-4.702053981	-4.5615442	2.7445E-05	-1.608577192	-0.6102793	0.24531308	-1.248626248	-1.51566424	0.030503
rb1	-0.962337721	-1.3427616	0.04541909	-0.629028841	-0.9174088	0.12094591	-1.67548007	-2.02552022	0.009429
rbbp4	-0.908285779	-0.9100184	0.12302165	-1.201365409	-1.6569109	0.02203379	-1.24715754	-1.24734394	0.056579
rbbp8	-2.278999538	-1.9380157	0.01153411	-0.535405417	-0.4932616	0.32117253	-3.343705646	-3.76072169	0.000173
rbm14	-1.302245937	-0.4423566	0.36111324	-1.0962076	-1.5748628	0.02661566	-2.190110164	-1.27047801	0.053644
recql	-0.490775461	-0.7859101	0.16371555	-0.839050312	-0.4439063	0.35982696	-0.546184811	-0.99078933	0.102143
recql4	-1.562673653	-1.2985545	0.05028581	-0.402605718	-0.7127293	0.19376291	-0.918201786	-0.67238953	0.212623
recql5	-3.493928118	-1.5441594	0.02856542	-0.923001255	-0.3506073	0.4460594	-2.097361702	-1.20849715	0.061873
rela	-2.363642398	-1.7156563	0.01924614	-0.140770165	-0.9470058	0.11297809	-1.26239305	-0.33489286	0.462495
rev1	-1.446625448	-0.9866108	0.10313098	-1.467188269	-0.7349806	0.18408541	-0.550722596	-0.1192774	0.759841
rev3l	-1.514467203	-0.617921	0.24103439	-2.014319025	-0.9911402	0.102061	-1.389299544	-0.54532927	0.284886
rexo2	-1.9174541	-0.7832419	0.16472448	-0.912619927	-0.310254	0.4894924	-2.63956837	-1.4002871	0.039784
rfc1	-1.018501409	-0.336452	0.46083767	-2.980361316	-2.2474898	0.00565601	-2.070863301	-1.73312175	0.018488
rfc2	-7.239427818	-3.1727878	0.00067176	-3.941378799	-3.3385092	0.00045866	-4.088888602	-1.93871179	0.011516
rfc3	-3.033529772	-2.0813274	0.00829225	-2.838272694	-4.7895263	1.6236E-05	-1.796262099	-2.27780812	0.005275

rfc4	-2.937627934	-2.6025691	0.00249707	-2.878063572	-3.4688171	0.00033977	-3.319113591	-3.51064866	0.000309
rfc5	-3.711062919	-2.1383522	0.0072719	-4.222339545	-3.4983776	0.00031741	-3.204499224	-1.91471333	0.01217
rfwd2	-1.544067704	-0.634597	0.2319546	-2.281988512	-1.2813981	0.05231206	-1.218018301	-0.54013332	0.288315
rint1	-0.571863931	-0.2652538	0.54293299	-0.577188664	-0.171231	0.67416935	-0.704560063	-0.26806416	0.539431
rnf168	-2.621616995	-0.9589625	0.10991007	-1.619218425	-0.915141	0.12157911	-1.021710458	-0.88962577	0.128936
rnf8	-1.113588234	-1.4378847	0.03648508	-1.466000282	-0.7495599	0.17800822	-2.620156263	-2.42957229	0.003719
rpa1	-1.444387705	-0.6252902	0.23697896	-1.405424439	-1.1435721	0.07185019	-0.831711115	-0.28301141	0.521181
rpa2	-1.797917482	-1.2940842	0.05080609	-2.557995331	-2.3733339	0.00423317	-3.256081348	-1.93084607	0.011726
rpa3	-1.107673087	-0.4840902	0.32802719	-1.35210917	-0.7723693	0.16890041	-1.783495846	-0.73835212	0.182662
rpa4	-0.417524227	-0.1854673	0.65242813	-1.389410393	-1.9358211	0.01159255	-0.682340692	-0.45684604	0.349264
rpain	-0.623999329	-0.4456518	0.35838363	-1.438993638	-1.0833213	0.0825427	-0.662768173	-0.9204915	0.12009
rpl13a	-3.800152378	-2.3960381	0.00401756	-2.571290093	-1.6973682	0.0200739	-0.926573837	-0.34654561	0.450251
rpl30	-4.625190211	-1.7638798	0.01722345	-1.473791132	-0.491341	0.32259603	-2.112592916	-1.07829431	0.083504
rprm	-0.656176187	-0.116847	0.76410493	-1.899410031	-1.1069663	0.07816884	-1.256732036	-0.78627748	0.163577
rps27a	-2.265449045	-1.0024789	0.09943083	-2.714253781	-1.6666107	0.02154712	-3.331643975	-2.05113716	0.008889
rrm1	-1.920894229	-1.1528859	0.07032571	-4.402987739	-3.8930804	0.00012791	-2.829789223	-2.04144653	0.00909
rrm2	-2.457456137	-1.3170194	0.04819263	-3.112365175	-5.5622291	2.7401E-06	-2.519544718	-1.41519442	0.038442
rrm2b	-0.730076873	-0.2357992	0.58103298	-2.327228759	-1.6000766	0.02511443	-1.533432805	-0.85510151	0.139604
ruvbl1	-4.482766574	-2.0630854	0.00864798	-1.296562773	-0.6868407	0.20566448	-3.088963256	-1.7637503	0.017229
ruvbl2	-3.970020681	-1.8423667	0.01437584	-1.537267974	-1.0646152	0.08617569	-1.599243718	-0.5535068	0.279572
sema4a	-1.057260753	-0.7010946	0.19902396	-0.946913498	-0.5757026	0.26564237	-1.161133052	-0.52293053	0.299964
sesn1	-1.233860689	-0.9667968	0.10794516	-1.822511472	-1.4274404	0.03737314	-0.971385545	-0.49409739	0.320555
setx	-0.535405632	-0.7023749	0.19843812	-0.754893328	-0.8542207	0.13988762	-1.006082903	-1.05461579	0.088183
sf3b3	-1.529306574	-1.0976116	0.07987087	-1.87384533	-0.9198086	0.12027944	-1.435205717	-0.61792099	0.241034
shfm1	-2.004339956	-1.4624665	0.03447732	-1.138287341	-1.2852766	0.05184697	-1.648773874	-1.32621245	0.047183
shisa5	-0.331813252	-0.1175125	0.76293486	-0.604571298	-0.6061175	0.24767521	-0.206292399	-0.32208878	0.476334
siah1	-1.469472721	-0.6742074	0.21173495	-2.059396432	-1.4048417	0.03936935	-2.036146052	-1.4040815	0.039438
sirt1	-1.14808893	-0.3433581	0.45356751	-0.654340628	-0.2056525	0.62279847	-1.107671904	-0.88458983	0.13044
slk	-1.814512729	-1.0385852	0.09149867	-0.810261868	-0.5816112	0.26205282	-1.234214299	-0.54843134	0.282858
slx4	-0.737472761	-0.2112142	0.61487352	-0.968425454	-0.4721929	0.33713751	-1.122273438	-0.80062906	0.15826
smc1a	-1.435539225	-0.6935193	0.20252598	-0.312330357	-0.8022817	0.15765882	-1.908894883	-0.91586186	0.121377
smc2	-4.376056663	-2.0284616	0.00936566	-1.695776128	-0.911715	0.12254201	-3.745617714	-1.8352859	0.014612
smc3	-2.640525339	-2.3606785	0.00435834	-2.554771991	-1.666236	0.02156572	-1.161211666	-0.30566219	0.494695
smc6	-1.878119646	-0.5749751	0.26608777	-1.167713483	-0.542823	0.28653454	-2.363670498	-5.63071347	2.34E-06
sod1	-0.898044284	-0.9081041	0.12356511	-1.233604562	-1.695737	0.02014944	-1.320796768	-1.20961731	0.061714
spo11	-0.878772586	-0.4932616	0.32117253	-2.110888118	-2.0215147	0.00951668	-1.083024187	-1.43631202	0.036617
ssbp1	-0.222244352	-0.208089	0.6193142	-1.389125763	-1.5545925	0.02788737	-1.037127419	-0.42758514	0.373607
steap3	-0.730012143	-0.4897685	0.32376623	-0.553631	-1.0069212	0.09841897	-0.779240536	-0.71008021	0.194948
sumo1	-1.178603961	-0.8389474	0.14489472	-0.760085818	-1.3974449	0.04004563	-1.6146078	-1.11318041	0.077058
supt3h	-1.696759906	-1.0161488	0.09634988	-0.641900442	-1.2714388	0.05352556	-1.31350741	-0.93917153	0.115035
tada3	-0.712442283	-0.828288	0.14849505	-0.169157121	-0.3288619	0.46896244	0.007180229	-0.02399421	0.94625
taf2	-1.072160386	-0.2479437	0.56501023	-2.306147907	-1.1071344	0.07813859	-1.501113642	-0.57865154	0.263845
taf5l	-1.500124128	-1.1420118	0.07210879	-1.335510148	-1.9539806	0.01111781	-0.59447607	-1.10880654	0.077838
tcea1	-0.663901336	-0.7416005	0.18130072	-1.443624029	-0.7054735	0.19702732	-1.063268028	-0.45277202	0.352556
tdg	-0.587132838	-0.8721154	0.13424083	-1.60513553	-0.7620636	0.1729563	-0.592646882	-0.50877719	0.309901
tdp1	-2.780268509	-1.122367	0.07544545	-1.044963706	-0.5755506	0.26573541	-1.221102955	-0.98139949	0.104376
terf1	-2.505693729	-2.0384485	0.00915275	-2.023494064	-1.1551103	0.06996644	-2.218767267	-1.72122821	0.019001
terf2	-0.296023336	-0.4014454	0.39678442	-0.703593552	-0.2748351	0.5310861	-0.70957725	-0.58161116	0.262053
tert	-0.935295787	-0.4923377	0.32185653	-1.297037631	-0.7717166	0.16915443	-1.939015084	-1.11585372	0.076585
tff2	-0.787421712	-1.0578215	0.08753434	-1.357429985	-0.7602433	0.17368276	-1.379893143	-0.97051513	0.107025
tgfb1	-1.467392433	-0.4245632	0.37621559	-1.515490576	-0.9768058	0.10548584	-1.286678548	-0.92854999	0.117883
timeless	-0.563400475	-0.094939	0.80363892	-2.156674596	-2.911103	0.00122715	-1.129944676	-0.53029337	0.294922
tipin	-1.167619984	-0.6528245	0.22242085	-2.28413701	-2.5439755	0.00285775	-0.983745057	-0.25078523	0.561325
tnfrsf10b	-0.321724469	-0.2167239	0.60712219	-0.475113373	-0.3752017	0.4215007	0.118071218	-0.3410803	0.455953
tnp1	-1.100374725	-1.4683866	0.03401053	-0.758148415	-0.3438002	0.45310602	-2.098013518	-1.70193019	0.019864
top1	-0.786882964	-0.3543708	0.44221062	-0.973207365	-0.3115027	0.48808711	-2.035262781	-0.88826115	0.129342
top2a	-0.477790228	-0.7524739	0.17681786	-0.726136676	-0.2456123	0.56805152	-0.485483662	-0.70976841	0.195088
topbp1	-3.232461304	-4.7105864	1.9472E-05	-1.255958924	-0.5623568	0.27393229	-2.094222826	-4.13988708	7.25E-05
tp53	-1.28968399	-1.4447978	0.03590891	-0.948795984	-0.6426821	0.22767634	-0.611379004	-0.3560607	0.440493
tp53bp1	-1.848691849	-1.7521969	0.01769307	-1.260964687	-1.0562184	0.08785807	-0.910515339	-1.09647588	0.08008
tp73	0.000655459	-0.9345193	0.11627349	-1.138233677	-0.4584728	0.34795833	-0.830639449	-1.1338453	0.073478
trex1	-0.268550418	-0.5529844	0.2799082	-0.928886944	-0.8710836	0.13456014	-1.016284921	-0.57959727	0.263271

trex2	-0.620786203	-0.739469	0.18219272	-1.335170074	-1.5054584	0.03122781	-0.661962741	-0.92362301	0.119228
triap1	-1.40686879	-1.252951	0.05585332	-2.770698213	-2.2442376	0.00569852	-0.853580324	-0.51774615	0.303567
trrap	-2.353469181	-1.9323543	0.01168546	-0.312829693	-0.1821807	0.65738426	-0.682807959	-0.2184851	0.604665
ttk	-0.684122461	-0.250058	0.56226625	-0.490018711	-0.2085461	0.61866272	-1.4744007	-0.61110733	0.244846
txn	-0.383473599	-0.5871976	0.25870353	-1.217135742	-0.4014454	0.39678442	-0.321293759	-0.06691591	0.857204
uba52	-4.30373312	-1.8812582	0.01314443	-0.650619784	-0.3847485	0.41233624	-2.89303663	-1.56236593	0.027393
ubb	-1.231834168	-0.7586047	0.17433931	-2.458550258	-1.2338575	0.05836366	-0.990842042	-1.16483227	0.068418
ube2a	-0.707173665	-1.0242789	0.09456297	-0.777683561	-1.3115217	0.04880658	-1.629000817	-0.83695364	0.145561
ube2b	-1.033377257	-0.4644655	0.3431899	-0.398438768	-0.6905714	0.20390534	-1.143081643	-0.51735885	0.303837
ube2i	-1.764459849	-0.7259664	0.18794622	-3.395357239	-3.0479502	0.00089547	-0.737081271	-0.19350427	0.640465
ube2n	-2.387329037	-1.474503	0.0335349	-1.297927041	-0.5388421	0.28917308	-3.881568826	-2.23051007	0.005882
ube2t	-1.811697876	-0.5905879	0.25669185	-0.325950072	-0.0965984	0.80057428	-1.98318714	-1.42584671	0.037511
ube2v1	-0.868722131	-1.7555763	0.01755593	-0.291189908	-0.562807	0.27364843	-1.10227026	-1.22120169	0.060089
ube2v2	-0.755502484	-0.1858604	0.6518379	-1.611235341	-0.8255037	0.14945015	-0.425427603	-0.20853245	0.618682
ung	-1.093882165	-0.8756939	0.13313924	-0.822225571	-0.2545174	0.55652229	-1.087509193	-1.12230372	0.075456
upf1	-2.487062438	-1.5559868	0.02779798	-2.751787024	-1.7324629	0.01851557	-1.055518281	-0.3675195	0.429023
usp1	-1.024246073	-0.3984828	0.39950035	-1.156904984	-0.5214617	0.30098044	-1.532203274	-0.61770106	0.241156
uvrag	-1.863234667	-1.5228992	0.02999859	-2.274334875	-1.85739	0.01388705	-2.312641527	-1.01415277	0.096794
vcp	-2.631617635	-2.9083429	0.00123497	-1.555665659	-1.3969574	0.0400906	-0.752757762	-0.26532685	0.542842
wdr33	-2.708128186	-2.181766	0.00658012	-2.973431285	-2.9416147	0.00114389	-2.668414578	-1.69514031	0.020177
wrap53	-1.845752685	-0.8290434	0.148237	-1.369333949	-1.1748323	0.06686021	-1.696451284	-1.0468422	0.089775
wrn	-1.472548426	-0.620486	0.23961502	-1.107800876	-0.6218663	0.23885465	-2.090469668	-1.76905992	0.017019
wrnip1	-1.124522663	-0.362825	0.43368564	-0.840058583	-0.2782132	0.52697116	-0.347512987	-0.08979651	0.813211
xab2	-2.664689047	-1.2487645	0.05639434	-0.412450346	-0.0825205	0.82695052	-2.035138029	-0.88425654	0.13054
xpa	-0.685939508	-1.2480253	0.05649041	-0.897804129	-0.3470623	0.44971531	-0.445811997	-0.26952431	0.53762
xpc	-1.156341152	-0.3684947	0.42806064	-0.573009856	-0.5715751	0.26817906	-0.109561302	-0.22231873	0.599351
xrcc1	-0.128066328	-0.2416243	0.57329182	-0.430134448	-0.1536701	0.70198834	-0.67306058	-1.05301382	0.088509
xrcc2	-1.323880193	-0.6416647	0.22821033	-0.315904344	-0.3534002	0.44320005	-1.127686842	-0.32734706	0.470601
xrcc3	-1.176429392	-0.2809453	0.52366639	-0.794419325	-0.3518426	0.44479247	-1.45811069	-0.48321888	0.328686
xrcc4	-0.551727552	-0.1149497	0.76745034	-0.130161256	-0.0712695	0.84865371	-0.504940787	-0.1980492	0.633798
xrcc5	-1.536155227	-1.7062317	0.01966837	-0.441799397	-0.3379476	0.45925337	-1.389403201	-0.90185774	0.125355
xrcc6	-1.30028468	-0.5053785	0.31233563	-0.438477105	-0.2937563	0.50844467	-0.596386192	-0.67292608	0.212361
xrcc6bp1	-1.580471879	-0.461368	0.34564633	-1.945566651	-0.9106081	0.12285473	-0.907864197	-0.20232781	0.627584
xrn2	-0.895702354	-0.8375465	0.14536286	-1.191050846	-0.9659146	0.10816467	-1.116438211	-1.94807183	0.01127
ybx1	-1.069213242	-0.8419978	0.14388059	-0.109194969	-0.2323331	0.58568877	-0.125133556	-0.37931434	0.417528
zak	-0.524881614	-0.1065235	0.78248584	-0.587373142	-0.1848615	0.65333886	-0.988201837	-0.56566943	0.271851
zw10	-2.170320011	-1.0730218	0.08452364	-1.101548672	-0.6029207	0.24950503	0.061630855	-0.03925215	0.913583
zwint	-1.586863324	-0.5233045	0.29970607	-0.95044799	-0.5856825	0.25960763	-1.854809159	-1.14011027	0.072425

Supplementary Table 5. Codelet. Correlation

cancer	total_number_patient	PTEN_MMS19_cor	CDH1_FAM96B_cor
ACC	76	1.000	0.919
BLCA	399	0.759	0.875
BRCA	981	0.716	0.941
CESC	272	0.748	0.940
CHOL	36	0.725	0.878
COAD	341	0.795	0.975
DLBC	37	0.756	0.837
ESCA	169	0.865	0.882
GBM	126	0.554	0.870
HNSC	487	0.884	0.953
KICH	65	0.974	1.000
KIRC	352	0.984	0.993
KIRP	271	0.918	1.000
LAML	115	0.658	-0.016
LGG	507	0.915	0.978
LIHC	348	0.841	0.956
LUAD	502	0.956	0.938
LUSC	464	0.712	0.930
MESO	82	0.778	0.844
OV	177	0.727	0.675
PAAD	152	0.927	1.000
PCPG	161	0.656	0.966
PRAD	479	0.467	0.868
READ	118	0.807	0.941
SARC	229	0.859	0.948
SKCM	363	0.830	0.938
STAD	383	0.625	0.916
TGCT	144	0.954	0.978
THCA	480	0.553	0.878
THYM	119	1.000	1.000
UCEC	507	0.762	0.805
UCS	56	0.926	0.638
UVM	80	1.000	0.966

Supplememtary Table 6. Statistical analysis for supplementary figure 6

Multiple Comparisons

2way ANOVA of DMSO: Normalized (to shLuc) metabolite concentrations

Within each row, compare columns (simple effects within rows)

Tukey's multiple comparisons test	Summary	Adjusted P Value
AMP		
SUM159_ShLuc-DMSO vs. SUM159_Sh-A3-DMSO	ns	0.6875
SUM159_ShLuc-DMSO vs. SUM159_ShMM-5-DMSO	ns	0.7925
SUM159_Sh-A3-DMSO vs. SUM159_ShMM-5-DMSO	ns	0.9831
ADP		
SUM159_ShLuc-DMSO vs. SUM159_Sh-A3-DMSO	ns	0.9427
SUM159_ShLuc-DMSO vs. SUM159_ShMM-5-DMSO	ns	0.8928
SUM159_Sh-A3-DMSO vs. SUM159_ShMM-5-DMSO	ns	0.9912
ATP		
SUM159_ShLuc-DMSO vs. SUM159_Sh-A3-DMSO	ns	>0.9999
SUM159_ShLuc-DMSO vs. SUM159_ShMM-5-DMSO	ns	0.9358
SUM159_Sh-A3-DMSO vs. SUM159_ShMM-5-DMSO	ns	0.9332
GMP		
SUM159_ShLuc-DMSO vs. SUM159_Sh-A3-DMSO	ns	0.8786
SUM159_ShLuc-DMSO vs. SUM159_ShMM-5-DMSO	ns	0.3905
SUM159_Sh-A3-DMSO vs. SUM159_ShMM-5-DMSO	ns	0.6867
GDP		
SUM159_ShLuc-DMSO vs. SUM159_Sh-A3-DMSO	ns	0.901
SUM159_ShLuc-DMSO vs. SUM159_ShMM-5-DMSO	ns	0.7173
SUM159_Sh-A3-DMSO vs. SUM159_ShMM-5-DMSO	ns	0.9374
GTP		
SUM159_ShLuc-DMSO vs. SUM159_Sh-A3-DMSO	ns	0.9522
SUM159_ShLuc-DMSO vs. SUM159_ShMM-5-DMSO	ns	0.9851
SUM159_Sh-A3-DMSO vs. SUM159_ShMM-5-DMSO	ns	0.8885
CMP		
SUM159_ShLuc-DMSO vs. SUM159_Sh-A3-DMSO	ns	0.7207
SUM159_ShLuc-DMSO vs. SUM159_ShMM-5-DMSO	ns	0.9881
SUM159_Sh-A3-DMSO vs. SUM159_ShMM-5-DMSO	ns	0.6289

CDP

SUM159_ShLuc-DMSO vs. SUM159_Sh-A3-DMSO	ns		0.1528
SUM159_ShLuc-DMSO vs. SUM159_ShMM-5-DMSO	ns		0.4745
SUM159_Sh-A3-DMSO vs. SUM159_ShMM-5-DMSO	ns		0.7662

CTP

SUM159_ShLuc-DMSO vs. SUM159_Sh-A3-DMSO	ns		0.6237
SUM159_ShLuc-DMSO vs. SUM159_ShMM-5-DMSO	ns		0.9941
SUM159_Sh-A3-DMSO vs. SUM159_ShMM-5-DMSO	ns		0.5583

IMP

SUM159_ShLuc-DMSO vs. SUM159_Sh-A3-DMSO	**		0.0037
SUM159_ShLuc-DMSO vs. SUM159_ShMM-5-DMSO	****	<0.0001	
SUM159_Sh-A3-DMSO vs. SUM159_ShMM-5-DMSO	*		0.0382

IDP

SUM159_ShLuc-DMSO vs. SUM159_Sh-A3-DMSO	ns	>0.9999	
SUM159_ShLuc-DMSO vs. SUM159_ShMM-5-DMSO	ns		0.9864
SUM159_Sh-A3-DMSO vs. SUM159_ShMM-5-DMSO	ns		0.9874

ITP

SUM159_ShLuc-DMSO vs. SUM159_Sh-A3-DMSO	ns		0.991
SUM159_ShLuc-DMSO vs. SUM159_ShMM-5-DMSO	ns		0.9999
SUM159_Sh-A3-DMSO vs. SUM159_ShMM-5-DMSO	ns		0.9886

m5UTP

SUM159_ShLuc-DMSO vs. SUM159_Sh-A3-DMSO	ns		0.4685
SUM159_ShLuc-DMSO vs. SUM159_ShMM-5-DMSO	ns		0.5489
SUM159_Sh-A3-DMSO vs. SUM159_ShMM-5-DMSO	ns		0.9906

TMP

SUM159_ShLuc-DMSO vs. SUM159_Sh-A3-DMSO	ns	>0.9999	
SUM159_ShLuc-DMSO vs. SUM159_ShMM-5-DMSO	ns		0.9223
SUM159_Sh-A3-DMSO vs. SUM159_ShMM-5-DMSO	ns		0.9221

TDP

SUM159_ShLuc-DMSO vs. SUM159_Sh-A3-DMSO	ns		0.0688
SUM159_ShLuc-DMSO vs. SUM159_ShMM-5-DMSO	ns		0.4905
SUM159_Sh-A3-DMSO vs. SUM159_ShMM-5-DMSO	ns		0.5203

TTP

SUM159_ShLuc-DMSO vs. SUM159_Sh-A3-DMSO	ns		0.9869
SUM159_ShLuc-DMSO vs. SUM159_ShMM-5-DMSO	ns		0.8742
SUM159_Sh-A3-DMSO vs. SUM159_ShMM-5-DMSO	ns		0.9385

UMP		
SUM159_ShLuc-DMSO vs. SUM159_Sh-A3-DMSO	ns	0.0977
SUM159_ShLuc-DMSO vs. SUM159_ShMM-5-DMSO	ns	0.1263
SUM159_Sh-A3-DMSO vs. SUM159_ShMM-5-DMSO	ns	0.992
UDP		
SUM159_ShLuc-DMSO vs. SUM159_Sh-A3-DMSO	ns	0.5237
SUM159_ShLuc-DMSO vs. SUM159_ShMM-5-DMSO	ns	0.9092
SUM159_Sh-A3-DMSO vs. SUM159_ShMM-5-DMSO	ns	0.7806
UTP		
SUM159_ShLuc-DMSO vs. SUM159_Sh-A3-DMSO	ns	0.3991
SUM159_ShLuc-DMSO vs. SUM159_ShMM-5-DMSO	ns	0.9259
SUM159_Sh-A3-DMSO vs. SUM159_ShMM-5-DMSO	ns	0.6267
XMP		
SUM159_ShLuc-DMSO vs. SUM159_Sh-A3-DMSO	ns	0.2002
SUM159_ShLuc-DMSO vs. SUM159_ShMM-5-DMSO	ns	0.8668
SUM159_Sh-A3-DMSO vs. SUM159_ShMM-5-DMSO	ns	0.4476
XDP		
SUM159_ShLuc-DMSO vs. SUM159_Sh-A3-DMSO	ns	0.9957
SUM159_ShLuc-DMSO vs. SUM159_ShMM-5-DMSO	ns	0.8699
SUM159_Sh-A3-DMSO vs. SUM159_ShMM-5-DMSO	ns	0.8249
XTP		
SUM159_ShLuc-DMSO vs. SUM159_Sh-A3-DMSO	ns	0.8667
SUM159_ShLuc-DMSO vs. SUM159_ShMM-5-DMSO	ns	0.2838
SUM159_Sh-A3-DMSO vs. SUM159_ShMM-5-DMSO	ns	0.5708
dAMP		
SUM159_ShLuc-DMSO vs. SUM159_Sh-A3-DMSO	*	0.0275
SUM159_ShLuc-DMSO vs. SUM159_ShMM-5-DMSO	ns	0.6739
SUM159_Sh-A3-DMSO vs. SUM159_ShMM-5-DMSO	ns	0.1895
dADP		
SUM159_ShLuc-DMSO vs. SUM159_Sh-A3-DMSO	ns	0.2414
SUM159_ShLuc-DMSO vs. SUM159_ShMM-5-DMSO	ns	0.9014
SUM159_Sh-A3-DMSO vs. SUM159_ShMM-5-DMSO	ns	0.465
dATP		
SUM159_ShLuc-DMSO vs. SUM159_Sh-A3-DMSO	ns	0.3836
SUM159_ShLuc-DMSO vs. SUM159_ShMM-5-DMSO	ns	0.9086

SUM159_Sh-A3-DMSO vs. SUM159_ShMM-5-DMSO	ns	0.6364
dCTP		
SUM159_ShLuc-DMSO vs. SUM159_Sh-A3-DMSO	ns	0.4432
SUM159_ShLuc-DMSO vs. SUM159_ShMM-5-DMSO	ns	0.9798
SUM159_Sh-A3-DMSO vs. SUM159_ShMM-5-DMSO	ns	0.3371
dGDP		
SUM159_ShLuc-DMSO vs. SUM159_Sh-A3-DMSO	ns	0.9548
SUM159_ShLuc-DMSO vs. SUM159_ShMM-5-DMSO	ns	0.9005
SUM159_Sh-A3-DMSO vs. SUM159_ShMM-5-DMSO	ns	0.9882
dGTP		
SUM159_ShLuc-DMSO vs. SUM159_Sh-A3-DMSO	ns	0.9488
SUM159_ShLuc-DMSO vs. SUM159_ShMM-5-DMSO	ns	0.3788
SUM159_Sh-A3-DMSO vs. SUM159_ShMM-5-DMSO	ns	0.5625
dIDP		
SUM159_ShLuc-DMSO vs. SUM159_Sh-A3-DMSO	ns	0.0929
SUM159_ShLuc-DMSO vs. SUM159_ShMM-5-DMSO	ns	0.5107
SUM159_Sh-A3-DMSO vs. SUM159_ShMM-5-DMSO	ns	0.5826
dITP		
SUM159_ShLuc-DMSO vs. SUM159_Sh-A3-DMSO	ns	0.5423
SUM159_ShLuc-DMSO vs. SUM159_ShMM-5-DMSO	ns	0.8234
SUM159_Sh-A3-DMSO vs. SUM159_ShMM-5-DMSO	ns	0.8889
dTDP		
SUM159_ShLuc-DMSO vs. SUM159_Sh-A3-DMSO	ns	0.2389
SUM159_ShLuc-DMSO vs. SUM159_ShMM-5-DMSO	ns	0.8445
SUM159_Sh-A3-DMSO vs. SUM159_ShMM-5-DMSO	ns	0.5347
dTTP		
SUM159_ShLuc-DMSO vs. SUM159_Sh-A3-DMSO	ns	>0.9999
SUM159_ShLuc-DMSO vs. SUM159_ShMM-5-DMSO	ns	0.7819
SUM159_Sh-A3-DMSO vs. SUM159_ShMM-5-DMSO	ns	0.779
dUMP		
SUM159_ShLuc-DMSO vs. SUM159_Sh-A3-DMSO	ns	0.2415
SUM159_ShLuc-DMSO vs. SUM159_ShMM-5-DMSO	ns	0.5366
SUM159_Sh-A3-DMSO vs. SUM159_ShMM-5-DMSO	*	0.0219
dUDP		
SUM159_ShLuc-DMSO vs. SUM159_Sh-A3-DMSO	****	<0.0001

SUM159_ShLuc-DMSO vs. SUM159_ShMM-5-DMSO	ns	0.7211
SUM159_Sh-A3-DMSO vs. SUM159_ShMM-5-DMSO	**	0.001
dUTP		
SUM159_ShLuc-DMSO vs. SUM159_Sh-A3-DMSO	ns	0.6015
SUM159_ShLuc-DMSO vs. SUM159_ShMM-5-DMSO	ns	0.3753
SUM159_Sh-A3-DMSO vs. SUM159_ShMM-5-DMSO	ns	0.9246
dXMP		
SUM159_ShLuc-DMSO vs. SUM159_Sh-A3-DMSO	**	0.0068
SUM159_ShLuc-DMSO vs. SUM159_ShMM-5-DMSO	****	<0.0001
SUM159_Sh-A3-DMSO vs. SUM159_ShMM-5-DMSO	ns	0.0683
dXDP		
SUM159_ShLuc-DMSO vs. SUM159_Sh-A3-DMSO	ns	0.7746
SUM159_ShLuc-DMSO vs. SUM159_ShMM-5-DMSO	ns	0.8224
SUM159_Sh-A3-DMSO vs. SUM159_ShMM-5-DMSO	ns	0.996
dXTP		
SUM159_ShLuc-DMSO vs. SUM159_Sh-A3-DMSO	ns	0.961
SUM159_ShLuc-DMSO vs. SUM159_ShMM-5-DMSO	ns	0.9973
SUM159_Sh-A3-DMSO vs. SUM159_ShMM-5-DMSO	ns	0.9784
cyclic-AMP		
SUM159_ShLuc-DMSO vs. SUM159_Sh-A3-DMSO	ns	0.3239
SUM159_ShLuc-DMSO vs. SUM159_ShMM-5-DMSO	ns	0.8194
SUM159_Sh-A3-DMSO vs. SUM159_ShMM-5-DMSO	ns	0.1061
cyclic-GMP		
SUM159_ShLuc-DMSO vs. SUM159_Sh-A3-DMSO	ns	0.4971
SUM159_ShLuc-DMSO vs. SUM159_ShMM-5-DMSO	ns	0.8039
SUM159_Sh-A3-DMSO vs. SUM159_ShMM-5-DMSO	ns	0.1866

Multiple Comparisons

2way ANOVA of Chk1i: Normalized (to shLuc) metabolite concentrations

Within each row, compare columns (simple effects within rows)

Tukey's multiple comparisons test	Summary	Adjusted P Value
AMP		
SUM159_ShLuc-Chk1i vs. SUM159_Sh-A3-Chk1i	ns	0.1559

SUM159_ShLuc-Chk1i vs. SUM159_ShMM-5-Chk1i	ns	0.5529
SUM159_Sh-A3-Chk1i vs. SUM159_ShMM-5-Chk1i	ns	0.6954
ADP		
SUM159_ShLuc-Chk1i vs. SUM159_Sh-A3-Chk1i	ns	0.4797
SUM159_ShLuc-Chk1i vs. SUM159_ShMM-5-Chk1i	ns	0.5724
SUM159_Sh-A3-Chk1i vs. SUM159_ShMM-5-Chk1i	ns	0.9877
ATP		
SUM159_ShLuc-Chk1i vs. SUM159_Sh-A3-Chk1i	ns	0.1648
SUM159_ShLuc-Chk1i vs. SUM159_ShMM-5-Chk1i	ns	0.4386
SUM159_Sh-A3-Chk1i vs. SUM159_ShMM-5-Chk1i	ns	0.822
GMP		
SUM159_ShLuc-Chk1i vs. SUM159_Sh-A3-Chk1i	ns	0.2513
SUM159_ShLuc-Chk1i vs. SUM159_ShMM-5-Chk1i	ns	0.4801
SUM159_Sh-A3-Chk1i vs. SUM159_ShMM-5-Chk1i	ns	0.9007
GDP		
SUM159_ShLuc-Chk1i vs. SUM159_Sh-A3-Chk1i	ns	0.1245
SUM159_ShLuc-Chk1i vs. SUM159_ShMM-5-Chk1i	ns	0.2797
SUM159_Sh-A3-Chk1i vs. SUM159_ShMM-5-Chk1i	ns	0.9014
GTP		
SUM159_ShLuc-Chk1i vs. SUM159_Sh-A3-Chk1i	ns	0.8023
SUM159_ShLuc-Chk1i vs. SUM159_ShMM-5-Chk1i	ns	0.9205
SUM159_Sh-A3-Chk1i vs. SUM159_ShMM-5-Chk1i	ns	0.565
CMP		
SUM159_ShLuc-Chk1i vs. SUM159_Sh-A3-Chk1i	ns	0.0734
SUM159_ShLuc-Chk1i vs. SUM159_ShMM-5-Chk1i	ns	0.7503
SUM159_Sh-A3-Chk1i vs. SUM159_ShMM-5-Chk1i	ns	0.3024
CDP		
SUM159_ShLuc-Chk1i vs. SUM159_Sh-A3-Chk1i	*	0.0294
SUM159_ShLuc-Chk1i vs. SUM159_ShMM-5-Chk1i	ns	0.5542
SUM159_Sh-A3-Chk1i vs. SUM159_ShMM-5-Chk1i	ns	0.2773
CTP		
SUM159_ShLuc-Chk1i vs. SUM159_Sh-A3-Chk1i	ns	0.3546
SUM159_ShLuc-Chk1i vs. SUM159_ShMM-5-Chk1i	ns	0.9819
SUM159_Sh-A3-Chk1i vs. SUM159_ShMM-5-Chk1i	ns	0.457
IMP		

SUM159_ShLuc-Chk1i vs. SUM159_Sh-A3-Chk1i	****	<0.0001
SUM159_ShLuc-Chk1i vs. SUM159_ShMM-5-Chk1i	****	<0.0001
SUM159_Sh-A3-Chk1i vs. SUM159_ShMM-5-Chk1i	ns	0.1766
IDP		
SUM159_ShLuc-Chk1i vs. SUM159_Sh-A3-Chk1i	ns	0.6243
SUM159_ShLuc-Chk1i vs. SUM159_ShMM-5-Chk1i	ns	0.7278
SUM159_Sh-A3-Chk1i vs. SUM159_ShMM-5-Chk1i	ns	0.9849
ITP		
SUM159_ShLuc-Chk1i vs. SUM159_Sh-A3-Chk1i	ns	0.2356
SUM159_ShLuc-Chk1i vs. SUM159_ShMM-5-Chk1i	ns	0.9771
SUM159_Sh-A3-Chk1i vs. SUM159_ShMM-5-Chk1i	ns	0.33
m5UTP		
SUM159_ShLuc-Chk1i vs. SUM159_Sh-A3-Chk1i	ns	0.3709
SUM159_ShLuc-Chk1i vs. SUM159_ShMM-5-Chk1i	ns	0.4567
SUM159_Sh-A3-Chk1i vs. SUM159_ShMM-5-Chk1i	ns	0.9875
TMP		
SUM159_ShLuc-Chk1i vs. SUM159_Sh-A3-Chk1i	ns	0.9942
SUM159_ShLuc-Chk1i vs. SUM159_ShMM-5-Chk1i	ns	0.8152
SUM159_Sh-A3-Chk1i vs. SUM159_ShMM-5-Chk1i	ns	0.8683
TDP		
SUM159_ShLuc-Chk1i vs. SUM159_Sh-A3-Chk1i	ns	0.7955
SUM159_ShLuc-Chk1i vs. SUM159_ShMM-5-Chk1i	ns	0.9172
SUM159_Sh-A3-Chk1i vs. SUM159_ShMM-5-Chk1i	ns	0.5521
TTP		
SUM159_ShLuc-Chk1i vs. SUM159_Sh-A3-Chk1i	ns	0.9856
SUM159_ShLuc-Chk1i vs. SUM159_ShMM-5-Chk1i	ns	0.8187
SUM159_Sh-A3-Chk1i vs. SUM159_ShMM-5-Chk1i	ns	0.7247
UMP		
SUM159_ShLuc-Chk1i vs. SUM159_Sh-A3-Chk1i	*	0.0202
SUM159_ShLuc-Chk1i vs. SUM159_ShMM-5-Chk1i	ns	0.632
SUM159_Sh-A3-Chk1i vs. SUM159_ShMM-5-Chk1i	ns	0.1738
UDP		
SUM159_ShLuc-Chk1i vs. SUM159_Sh-A3-Chk1i	ns	0.678
SUM159_ShLuc-Chk1i vs. SUM159_ShMM-5-Chk1i	ns	0.9999
SUM159_Sh-A3-Chk1i vs. SUM159_ShMM-5-Chk1i	ns	0.6679

UTP		
SUM159_ShLuc-Chk1i vs. SUM159_Sh-A3-Chk1i	ns	0.2815
SUM159_ShLuc-Chk1i vs. SUM159_ShMM-5-Chk1i	ns	0.9745
SUM159_Sh-A3-Chk1i vs. SUM159_ShMM-5-Chk1i	ns	0.3919
XMP		
SUM159_ShLuc-Chk1i vs. SUM159_Sh-A3-Chk1i	ns	0.0767
SUM159_ShLuc-Chk1i vs. SUM159_ShMM-5-Chk1i	ns	0.9359
SUM159_Sh-A3-Chk1i vs. SUM159_ShMM-5-Chk1i	*	0.0328
XDP		
SUM159_ShLuc-Chk1i vs. SUM159_Sh-A3-Chk1i	ns	0.9932
SUM159_ShLuc-Chk1i vs. SUM159_ShMM-5-Chk1i	ns	0.9217
SUM159_Sh-A3-Chk1i vs. SUM159_ShMM-5-Chk1i	ns	0.8731
XTP		
SUM159_ShLuc-Chk1i vs. SUM159_Sh-A3-Chk1i	ns	0.9793
SUM159_ShLuc-Chk1i vs. SUM159_ShMM-5-Chk1i	ns	0.6036
SUM159_Sh-A3-Chk1i vs. SUM159_ShMM-5-Chk1i	ns	0.7253
dAMP		
SUM159_ShLuc-Chk1i vs. SUM159_Sh-A3-Chk1i	*	0.0431
SUM159_ShLuc-Chk1i vs. SUM159_ShMM-5-Chk1i	ns	0.3709
SUM159_Sh-A3-Chk1i vs. SUM159_ShMM-5-Chk1i	ns	0.5296
dADP		
SUM159_ShLuc-Chk1i vs. SUM159_Sh-A3-Chk1i	*	0.0258
SUM159_ShLuc-Chk1i vs. SUM159_ShMM-5-Chk1i	ns	0.8023
SUM159_Sh-A3-Chk1i vs. SUM159_ShMM-5-Chk1i	ns	0.118
dATP		
SUM159_ShLuc-Chk1i vs. SUM159_Sh-A3-Chk1i	ns	0.3546
SUM159_ShLuc-Chk1i vs. SUM159_ShMM-5-Chk1i	ns	0.9193
SUM159_Sh-A3-Chk1i vs. SUM159_ShMM-5-Chk1i	ns	0.5857
dCTP		
SUM159_ShLuc-Chk1i vs. SUM159_Sh-A3-Chk1i	ns	0.1438
SUM159_ShLuc-Chk1i vs. SUM159_ShMM-5-Chk1i	ns	0.9926
SUM159_Sh-A3-Chk1i vs. SUM159_ShMM-5-Chk1i	ns	0.1804
dGDP		
SUM159_ShLuc-Chk1i vs. SUM159_Sh-A3-Chk1i	ns	0.336
SUM159_ShLuc-Chk1i vs. SUM159_ShMM-5-Chk1i	ns	0.989
SUM159_Sh-A3-Chk1i vs. SUM159_ShMM-5-Chk1i	ns	0.4127

dGTP		
SUM159_ShLuc-Chk1i vs. SUM159_Sh-A3-Chk1i	ns	0.2918
SUM159_ShLuc-Chk1i vs. SUM159_ShMM-5-Chk1i	ns	0.8325
SUM159_Sh-A3-Chk1i vs. SUM159_ShMM-5-Chk1i	ns	0.6241
dIDP		
SUM159_ShLuc-Chk1i vs. SUM159_Sh-A3-Chk1i	ns	0.155
SUM159_ShLuc-Chk1i vs. SUM159_ShMM-5-Chk1i	ns	0.6206
SUM159_Sh-A3-Chk1i vs. SUM159_ShMM-5-Chk1i	ns	0.6263
dITP		
SUM159_ShLuc-Chk1i vs. SUM159_Sh-A3-Chk1i	ns	0.2728
SUM159_ShLuc-Chk1i vs. SUM159_ShMM-5-Chk1i	ns	0.9375
SUM159_Sh-A3-Chk1i vs. SUM159_ShMM-5-Chk1i	ns	0.4534
dTDP		
SUM159_ShLuc-Chk1i vs. SUM159_Sh-A3-Chk1i	ns	0.2069
SUM159_ShLuc-Chk1i vs. SUM159_ShMM-5-Chk1i	ns	0.9982
SUM159_Sh-A3-Chk1i vs. SUM159_ShMM-5-Chk1i	ns	0.229
dTTP		
SUM159_ShLuc-Chk1i vs. SUM159_Sh-A3-Chk1i	ns	0.9151
SUM159_ShLuc-Chk1i vs. SUM159_ShMM-5-Chk1i	ns	0.8268
SUM159_Sh-A3-Chk1i vs. SUM159_ShMM-5-Chk1i	ns	0.5848
dUMP		
SUM159_ShLuc-Chk1i vs. SUM159_Sh-A3-Chk1i	ns	0.4907
SUM159_ShLuc-Chk1i vs. SUM159_ShMM-5-Chk1i	ns	0.4602
SUM159_Sh-A3-Chk1i vs. SUM159_ShMM-5-Chk1i	ns	0.0544
dUDP		
SUM159_ShLuc-Chk1i vs. SUM159_Sh-A3-Chk1i	ns	0.2026
SUM159_ShLuc-Chk1i vs. SUM159_ShMM-5-Chk1i	ns	0.6246
SUM159_Sh-A3-Chk1i vs. SUM159_ShMM-5-Chk1i	*	0.0245
dUTP		
SUM159_ShLuc-Chk1i vs. SUM159_Sh-A3-Chk1i	ns	0.3001
SUM159_ShLuc-Chk1i vs. SUM159_ShMM-5-Chk1i	ns	0.881
SUM159_Sh-A3-Chk1i vs. SUM159_ShMM-5-Chk1i	ns	0.124
dXMP		
SUM159_ShLuc-Chk1i vs. SUM159_Sh-A3-Chk1i	***	0.0005
SUM159_ShLuc-Chk1i vs. SUM159_ShMM-5-Chk1i	****	<0.0001

SUM159_Sh-A3-Chk1i vs. SUM159_ShMM-5-Chk1i	ns	0.3165
dXDP		
SUM159_ShLuc-Chk1i vs. SUM159_Sh-A3-Chk1i	ns	0.4439
SUM159_ShLuc-Chk1i vs. SUM159_ShMM-5-Chk1i	ns	0.5426
SUM159_Sh-A3-Chk1i vs. SUM159_ShMM-5-Chk1i	ns	0.9856
dXTP		
SUM159_ShLuc-Chk1i vs. SUM159_Sh-A3-Chk1i	ns	0.9996
SUM159_ShLuc-Chk1i vs. SUM159_ShMM-5-Chk1i	ns	0.999
SUM159_Sh-A3-Chk1i vs. SUM159_ShMM-5-Chk1i	ns	0.9973
cyclic-AMP		
SUM159_ShLuc-Chk1i vs. SUM159_Sh-A3-Chk1i	ns	0.2391
SUM159_ShLuc-Chk1i vs. SUM159_ShMM-5-Chk1i	ns	0.6573
SUM159_Sh-A3-Chk1i vs. SUM159_ShMM-5-Chk1i	*	0.0359
cyclic-GMP		
SUM159_ShLuc-Chk1i vs. SUM159_Sh-A3-Chk1i	**	0.0034
SUM159_ShLuc-Chk1i vs. SUM159_ShMM-5-Chk1i	ns	0.1754
SUM159_Sh-A3-Chk1i vs. SUM159_ShMM-5-Chk1i	ns	0.2881

Uncropped and unprocessed scans of western blots

Figure 2a

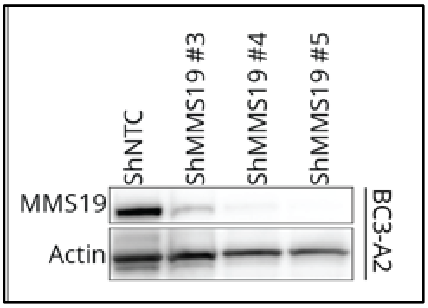

MMS19

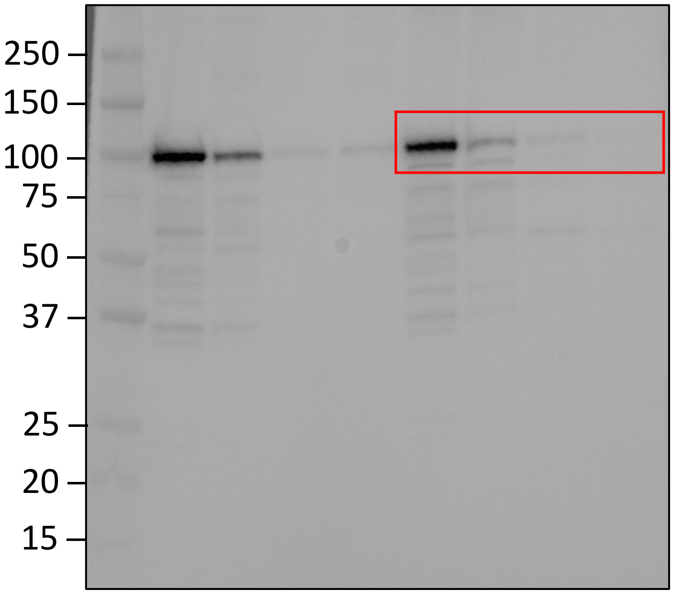

β-Actin

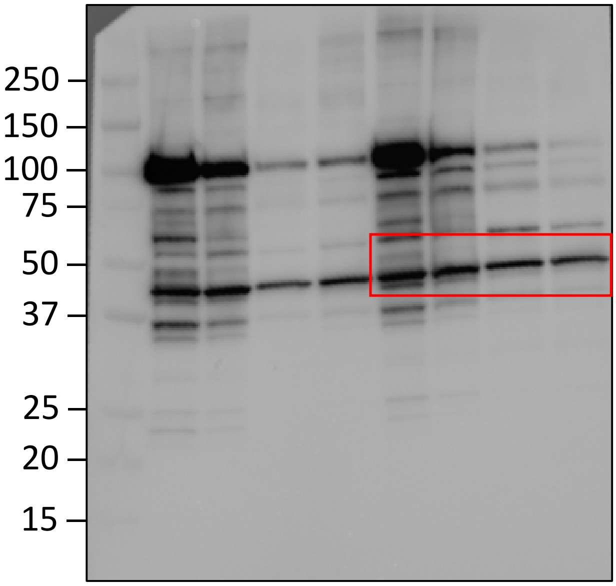

Figure 2c

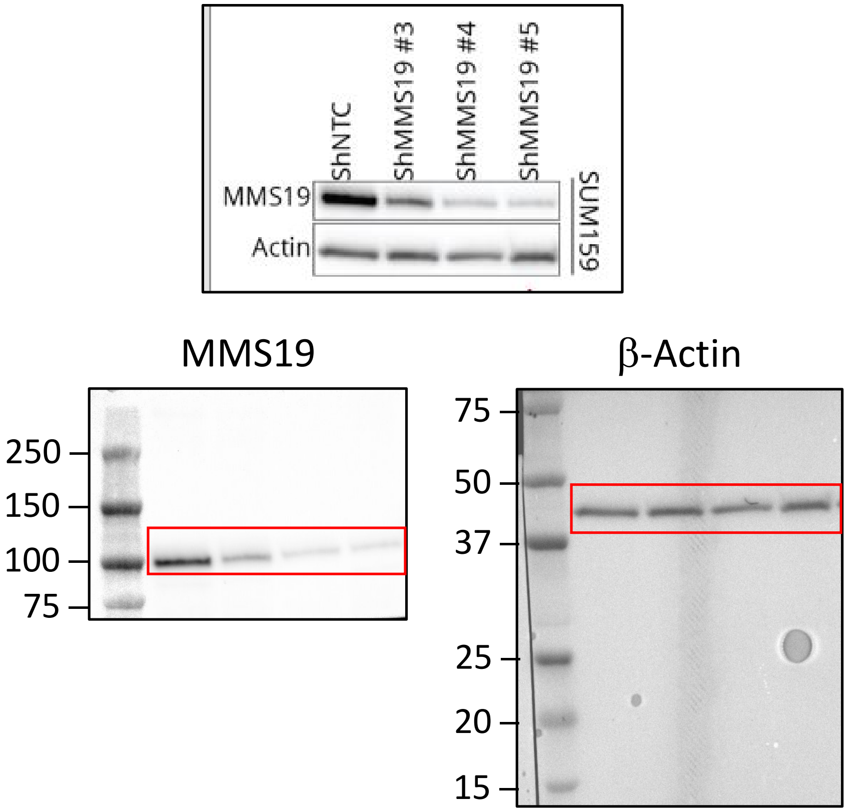

Figure 4a

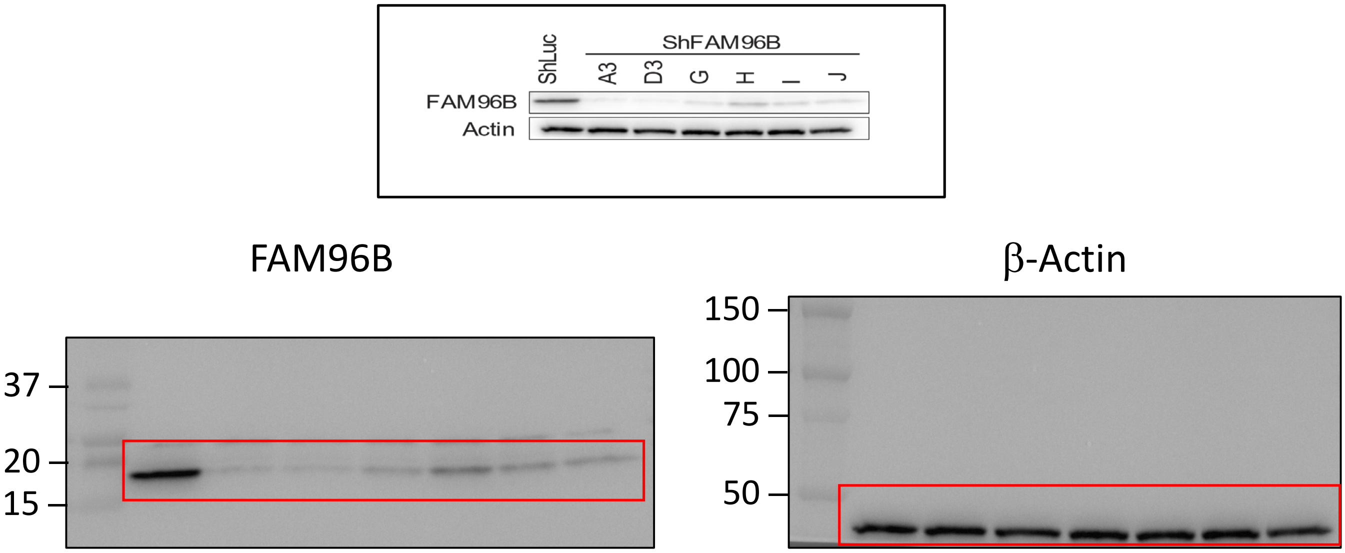

Supplementary Figure 1a

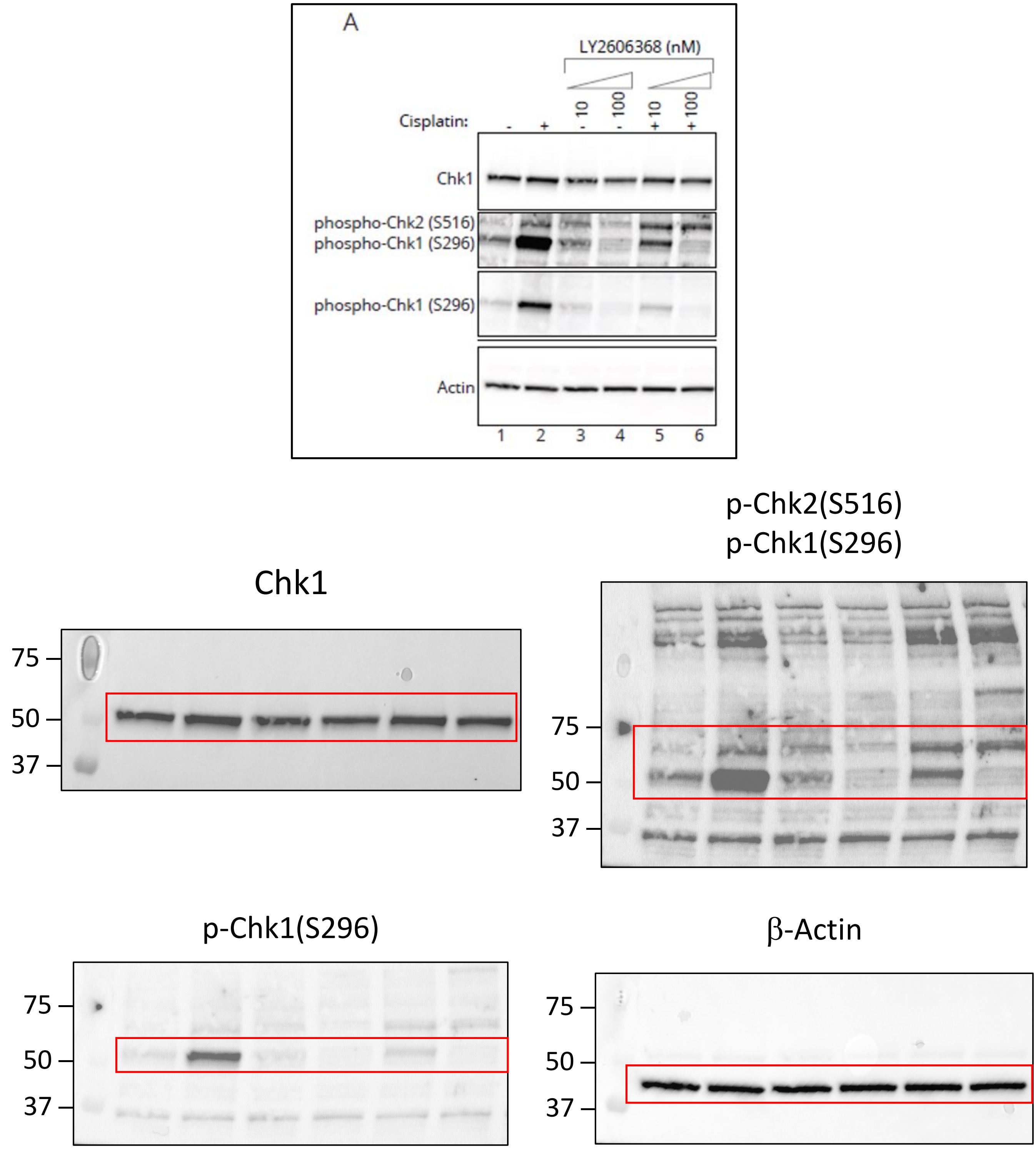

Supplementary Figure 3a

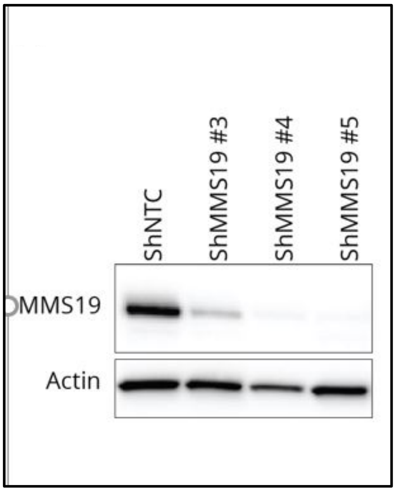

MMS19

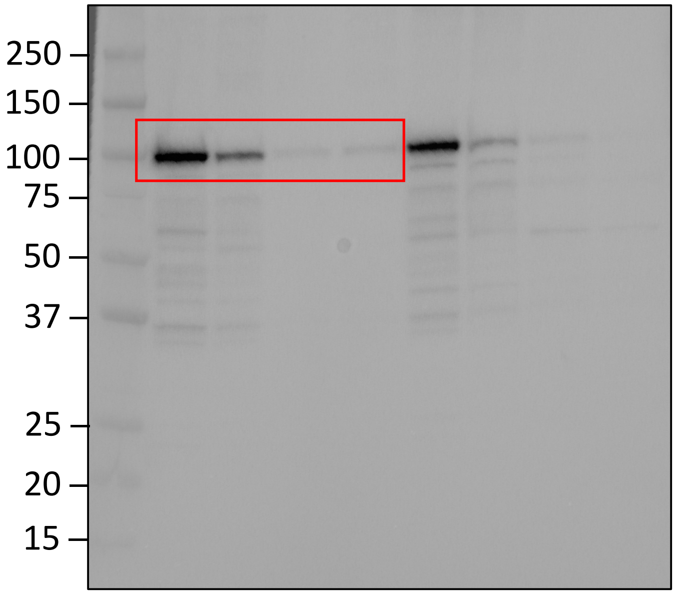

β-Actin

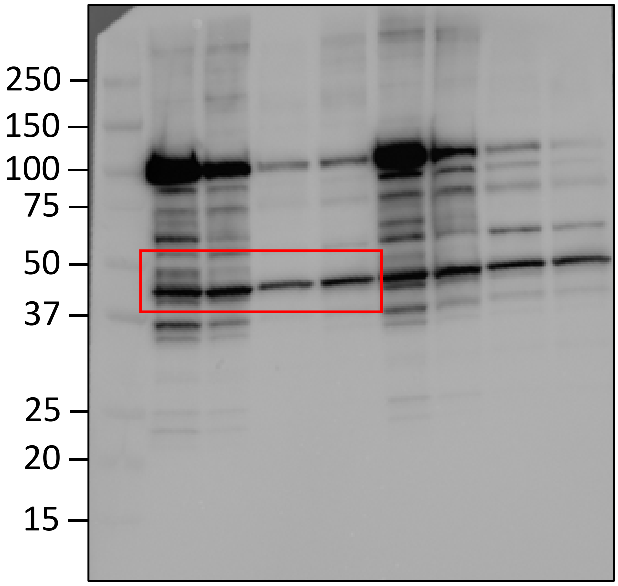

Supplementary Figure 5

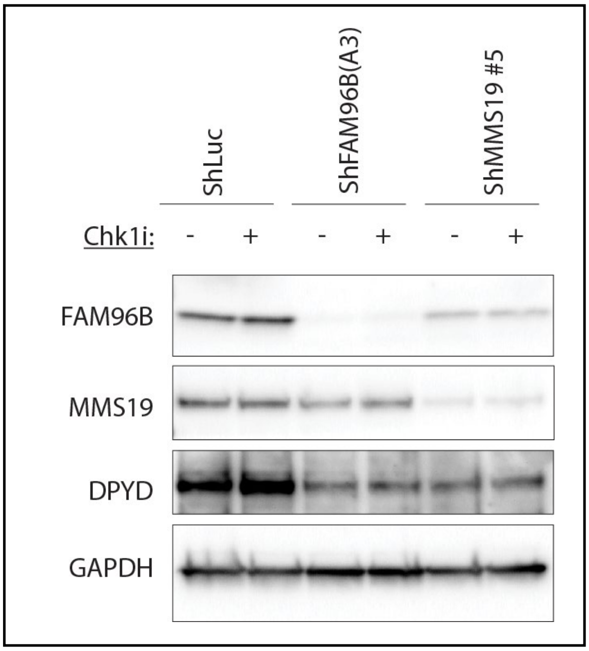

FAM96B

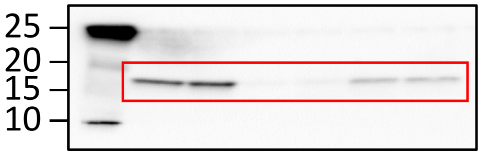

MMS19

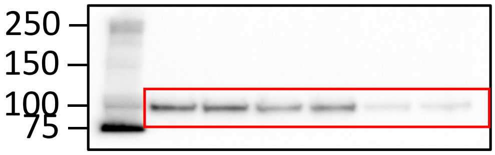

DPYD

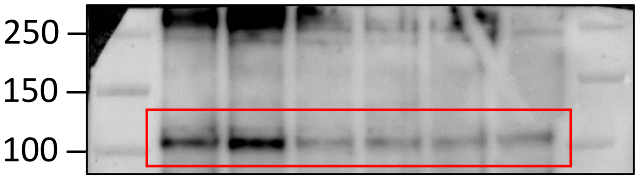

GAPDH

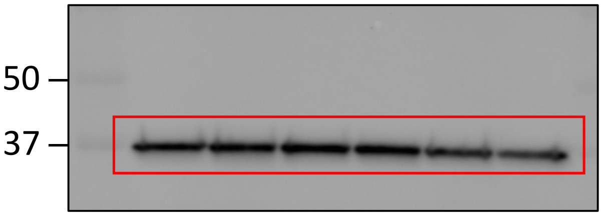

Supplement: Supplementary file 1 — Supplemental Material [file 41523_2021_353_MOESM1_ESM.pdf]
