# Supplementary material for: The cytosolic iron–sulfur cluster assembly (CIA) pathway is required for replication stress tolerance of cancer cells to Chk1 and ATR inhibitors
Source: NPJ Breast Cancer. 2021 Dec 2;7:152. doi: 10.1038/s41523-021-00353-2 (PMC8639742; doi:10.1038/s41523-021-00353-2)
Supplement: Supplementary file 2 — Reporting Summary [file 41523_2021_353_MOESM2_ESM.pdf]

nature portfolio

Corresponding author(s): Helen Piwnica-Worms

Last updated by author(s): Oct 27, 2021

Reporting Summary

Nature Portfolio wishes to improve the reproducibility of the work that we publish. This form provides structure for consistency and transparency in reporting. For further information on Nature Portfolio policies, see our Editorial Policies and the Editorial Policy Checklist.

Statistics

For all statistical analyses, confirm that the following items are present in the figure legend, table legend, main text, or Methods section.

n/a	Confirmed	
☐	☒	The exact sample size (n) for each experimental group/condition, given as a discrete number and unit of measurement
☐	☒	A statement on whether measurements were taken from distinct samples or whether the same sample was measured repeatedly
☐	☒	The statistical test(s) used AND whether they are one- or two-sided<br>*Only common tests should be described solely by name; describe more complex techniques in the Methods section.*
☒	☐	A description of all covariates tested
☐	☒	A description of any assumptions or corrections, such as tests of normality and adjustment for multiple comparisons
☐	☒	A full description of the statistical parameters including central tendency (e.g. means) or other basic estimates (e.g. regression coefficient) AND variation (e.g. standard deviation) or associated estimates of uncertainty (e.g. confidence intervals)
☐	☒	For null hypothesis testing, the test statistic (e.g. F, t, r) with confidence intervals, effect sizes, degrees of freedom and P value noted<br>*Give P values as exact values whenever suitable.*
☒	☐	For Bayesian analysis, information on the choice of priors and Markov chain Monte Carlo settings
☒	☐	For hierarchical and complex designs, identification of the appropriate level for tests and full reporting of outcomes
☒	☐	Estimates of effect sizes (e.g. Cohen's d, Pearson's r), indicating how they were calculated

Our web collection on statistics for biologists contains articles on many of the points above.

Software and code

Policy information about availability of computer code

Data collection	No software was used to collect data
Data analysis	GraphPad Prism 8 for Windows 64-bit Version 8.0.0(224), FCS Express, TIBCO Spotfire, Incucyte ZOOM, Sciex MultiQuant

For manuscripts utilizing custom algorithms or software that are central to the research but not yet described in published literature, software must be made available to editors and reviewers. We strongly encourage code deposition in a community repository (e.g. GitHub). See the Nature Portfolio guidelines for submitting code & software for further information.

Data

Policy information about availability of data

All manuscripts must include a data availability statement. This statement should provide the following information, where applicable:
- Accession codes, unique identifiers, or web links for publicly available datasets
- A description of any restrictions on data availability
- For clinical datasets or third party data, please ensure that the statement adheres to our policy

The data supporting the findings of this study are available within the paper and its supplementary information files.

Field-specific reporting

Please select the one below that is the best fit for your research. If you are not sure, read the appropriate sections before making your selection.

☒ Life sciences ☐ Behavioural & social sciences ☐ Ecological, evolutionary & environmental sciences

For a reference copy of the document with all sections, see nature.com/documents/nr-reporting-summary-flat.pdf

Life sciences study design

All studies must disclose on these points even when the disclosure is negative.

Sample size	All cell culture experiments included triplicate wells to account for technical variation. These experiments were repeated in triplicate to determine the reproducibility of the findings.
Data exclusions	No data exclusions.
Replication	All drug studies were performed in triplicate.
Randomization	No randomization was needed in this study. For cell culture experiments, all experimental conditions were plated out from the same cell stock to ensure a common starting point for the experiment.
Blinding	Blinding was not possible for these experiments, as the investigators were required to know which wells received which treatment.

Reporting for specific materials, systems and methods

We require information from authors about some types of materials, experimental systems and methods used in many studies. Here, indicate whether each material, system or method listed is relevant to your study. If you are not sure if a list item applies to your research, read the appropriate section before selecting a response.

Materials & experimental systems

n/a	Involved in the study	
☐	☒	Antibodies
☐	☒	Eukaryotic cell lines
☒	☐	Palaeontology and archaeology
☒	☐	Animals and other organisms
☒	☐	Human research participants
☒	☐	Clinical data
☒	☐	Dual use research of concern

Methods

n/a	Involved in the study	
☒	☐	ChIP-seq
☐	☒	Flow cytometry
☒	☐	MRI-based neuroimaging

Antibodies

Antibodies used	DPYD Monoclonal Antibody (7D4), ThermoFisher Scientific Cat # H00001806-M01; Phospho-Chk2 (Ser516) Polyclonal Antibody, CST Cat #2669; Chk1 (2G1D5) Mouse mAb, CST Cat #2360; GAPDH (D16H11) XP Rabbit mAb (HRP Conjugate), CST Cat #8884; β-Actin (D6A8) Rabbit mAb (HRP Conjugate), CST Cat #12620; Phospho-Chk1 (S296) Rabbit mAb, abcam Cat #ab79758; MMS19 Polyclonal Antibody, Proteintech Cat #16015-1-AP; FAM96B Polyclonal Antibody, Proteintech Cat #20108-1-AP.
Validation	DYPD: Antibody reactivity against cell lysate and recombinant protein for WB. It has also been used for IF and ELISA. Quality control test: Antibody Reactive Against Recombinant Protein.<br>Phospho-Chk2 (Ser516) Antibody detects endogenous levels of Chk2 only when phosphorylated at serine 516. The antibody does not cross-react with Chk2 phosphorylated at other sites.<br>Chk1 (2G1D5) Mouse mAb recognizes endogenous levels of total Chk1 protein.<br>GAPDH (D16H11) XP® Rabbit mAb (HRP Conjugate) detects endogenous levels of total GAPDH protein.<br>β-Actin (D6A8) Rabbit mAb (HRP Conjugate) recognizes endogenous levels of total β-actin protein. Due to the high sequence identity between the cytoplasmic actin isoforms, β-actin and cytoplasmic γ-actin, this antibody may cross-react with cytoplasmic γ-actin. It does not cross-react with α-skeletal, α-cardiac, α-vascular smooth, or γ-enteric smooth muscle isoforms.<br>Chk1 p-S296: The antibody only detects Chk1 phosphorylated at Serine 296.<br>MMS19: detects endogenous levels of total MMS19 protein.<br>FAM96B: detects endogenous levels of total FAM96B protein.

Eukaryotic cell lines

Policy information about cell lines

Cell line source(s)	BC3-A2 cells from Washington University in St. Louis; SUM159 cells from MD Anderson Cancer Center in Houston; and MDA-MB-231 cells from ATCC
Authentication	Cell lines purchased from ATCC are accompanied by the authentication document as provided by ATCC verifying the identity according to the short tandem repeats (STR) profile. In our laboratory, cell lines undergo follow-up STR verification before use by our institutional Characterized Cell Line Core.
Mycoplasma contamination	All cells used in our experiments tested negative for mycoplasma by PCR.
Commonly misidentified lines (See ICLAC register)	No commonly misidentified lines were used.

Flow Cytometry

Plots

Confirm that:

☒ The axis labels state the marker and fluorochrome used (e.g. CD4-FITC).

☒ The axis scales are clearly visible. Include numbers along axes only for bottom left plot of group (a 'group' is an analysis of identical markers).

☒ All plots are contour plots with outliers or pseudocolor plots.

☒ A numerical value for number of cells or percentage (with statistics) is provided.

Methodology

Sample preparation	Cells were treated with vehicle or Chk1i for 24 hours, then harvested and fixed in 70% ethanol, stained with DAPI and LIVE/DEAD™ Fixable Near-IR Dead Cell Stain Kit, for 633 or 635 nm excitation
Instrument	Data was collected using a Beckman Coulter Gallios, 3 laser configuration.
Software	The fraction of cells in G1, S, and G2/M was quantitated using FCS Express.
Cell population abundance	No cell sorting was performed.
Gating strategy	For DNA content analysis, cells were gated based on DAPI vs DAPI Peak, then analyzed for DAPI signal. For cell cycle analysis, cells were gated based on FSC vs LIVE/DEAD marker, then analyzed for DAPI signal

☒ Tick this box to confirm that a figure exemplifying the gating strategy is provided in the Supplementary Information.
